# Population genomic diversity and structure in the golden bandicoot: a history of isolation, extirpation, and conservation

**DOI:** 10.1038/s41437-023-00653-2

**Published:** 2023-10-08

**Authors:** Kate Rick, Margaret Byrne, Skye Cameron, Steve J. B. Cooper, Judy Dunlop, Brydie Hill, Cheryl Lohr, Nicola J. Mitchell, Craig Moritz, Kenny J. Travouillon, Brenton von Takach, Kym Ottewell

**Affiliations:** 1 School of Biological Sciences, The University of Western Australia, Crawley, WA 6009 Australia; 2 Biodiversity and Conservation Science, Department of Biodiversity, Conservation and Attractions, Kensington, WA 6152 Australia; 3 Australian Wildlife Conservancy, Level 2 322 Hay Street, Subiaco, WA 6008 Australia; 4 South Australian Museum, North Terrace, Adelaide, SA 5000 Australia; 5 Department of Ecology and Evolutionary Biology, Environment Institute, School of Biological Sciences, The University of Adelaide, Adelaide, SA 5005 Australia; 6 School of Agriculture and Environment, The University of Western Australia, Crawley, WA 6009 Australia; 7 Flora and Fauna Division, Department of Environment, Parks and Water Security, Northern Territory Government, Darwin, NT Australia; 8 Division of Ecology and Evolution, Research School of Biology, The Australian National University, Canberra, ACT Australia; 9 Collections and Research, Western Australian Museum, Welshpool, WA 6106 Australia; 10 School of Molecular and Life Sciences, Curtin University, Perth, WA 6102 Australia

**Keywords:** Ecology, Genetics

## Abstract

Using genetic information to develop and implement conservation programs is vital for maintaining biodiversity and ecosystem resilience. Evaluation of the genetic variability within and among remnant populations can inform management of both natural and translocated populations to maximise species’ adaptive potential, mitigate negative impacts of inbreeding, and subsequently minimise risk of extinction. Here we use reduced representation sequencing to undertake a genetic assessment of the golden bandicoot (*I*s*oodon auratus*), a threatened marsupial endemic to Australia. The currently recognised taxon consists of three subspecies distributed among multiple natural and translocated populations. After confirming the genetic distinctiveness of *I. auratus* from two closely related taxa, *I. fusciventer* and *I. macrourus*, we identified four genetic clusters within *I. auratus*. These clusters exhibited substantial genetic differentiation (pairwise F_ST_ values ranging from 0.18 to 0.65, pairwise D_XY_ ranging from 0.1 to 0.168), reflecting long-term isolation of some populations on offshore islands and the influence of genetic drift. Mainland natural populations in the Kimberley region had the highest genetic diversity and the largest contribution to overall allelic and gene diversity compared to both natural and translocated island populations. A population translocated to Guluwuru Island in the Northern Territory had the lowest genetic diversity. Our data suggest that island populations can appear genetically unique due to genetic drift and this needs to be taken into account when considering genetic diversity in conservation efforts to maintain overall genetic diversity of the species. We effectively demonstrate how genomic information can guide practical conservation planning, especially when declining species are represented by multiple isolated populations.

## Introduction

Genetic diversity is a fundamental element of biodiversity and helps drive ecosystem resilience, stability, and services (Raffard et al. 2019; Reynolds et al. 2012). Low genetic diversity can increase the risk of extinction, and management actions to maintain or increase genetic variation are increasingly advocated for in the conservation of threatened and keystone species (Hoban et al. 2021; Hoffmann et al. 2015; Weeks et al. 2011). Despite a previous emphasis on demographic concerns in threatened species management, the integration of genetic considerations into species conservation has grown substantially (Haig et al. 2016; Hoban et al. 2021; Kershaw et al. 2022; McDonald et al. 2015; Ottewell and Byrne 2022). Leveraging genetic information is pivotal for addressing the loss of genetic variation and subsequent adaptive potential, mitigating inbreeding effects, and predicting species’ resilience to demographic, environmental and/or genetic stochasticity (Frankham et al. 2017; Hoban et al. 2021; Ralls et al. 2018). Knowledge of genetic variability of remnant populations provides information as a baseline for ongoing management (e.g. von Takach et al. 2023), identifying populations requiring supplementation (Pacioni et al. 2020; Undin et al. 2021; Weeks et al. 2017; White et al. 2020), selecting optimal sources for translocations (Robinson et al. 2021; Weeks et al. 2011), and evaluating management outcomes (Ottewell et al. 2014; Rick et al. 2019; Thavornkanlapachai et al. 2019). International guidelines now advocate protecting at least 90% of genetic diversity within species (Hoban et al. 2020, 2021), but it is not always clear how best this can be achieved in relation to threatened species management.

Remnant populations, particularly those found on islands, often exhibit high levels of genetic and phenotypic divergence (Robertson et al. 2014). Evidence of strong population structure, which can arise rapidly in small and fragmented populations, can lead conservation managers to treat these as independent units, which can unintentionally reinforce the effects of isolation and subsequent extinction risk (Weeks et al. 2016). While population-specific management may be useful for populations that exhibit adaptive differences, such as those termed Evolutionarily Significant Units (ESUs) (Casacci et al. 2014; Coates et al. 2018; Moritz 1994), it is important to consider the degree to which observed population divergence is adaptive versus the result of stochastic processes (e.g. founder effects, genetic drift, etc.). Further, infraspecific units (e.g. subspecies) are often delimited based on morphology and do not always reflect underlying population genetic structure, which may overinflate the evolutionary significance of the infraspecific unit relative to other populations and impede species-level conservation actions (Robertson et al. 2014; Wolf and Ellegren 2017). This recognition is leading to a paradigm shift, with improved understanding that the loss of genetic diversity, the accumulation of genetic load and inbreeding are more proximal threats to species persistence than the risks of mixing genetically distinct populations (Frankham et al. 2017; Hoffmann et al. 2021; Ralls et al. 2018; Weeks et al. 2016). Consequently, there is a growing appreciation among conservation managers to prioritise management of these threats rather than being prescriptive in maintaining separate management of fragmented populations.

Australia faces a severe mammal extinction crisis (Woinarski et al. 2015, 2019), largely as a result of the introduction of feral cats (*Felix catus*) and red foxes (*Vulpes vulpes*) (Woinarski et al. 2015). The conservation of surviving mammals often relies heavily on insurance populations on islands or in predator-free fenced reserve “havens” (Legge et al. 2018). The latter approach has proven to be highly successful in establishing populations of Australian mammal species extirpated from their historic mainland ranges (Legge et al. 2018; Woinarski et al. 2023). Despite the popularity of utilising islands as sources for translocations this can also lead to many conservation challenges. The physical isolation of islands and fenced areas can subject these populations to founder effects, genetic bottlenecks, small population sizes, and limited dispersal, leading to the potential erosion of genetic diversity and demographic stochasticity. Thus, it is necessary to prioritise genetic management of remnant populations and to include genetic information in strategic decision-making for species’ recovery.

**Fig. 1:**
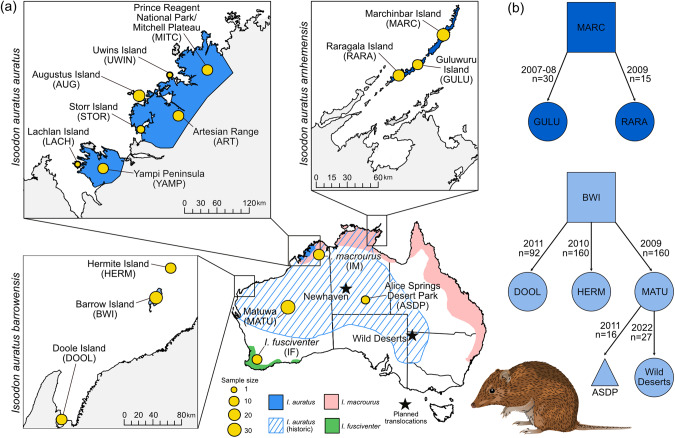
Distribution and translocation history of *Isoodon auratus*. (**a**) Distribution of *I. auratus* (golden bandicoot*), I. macrourus* (northern brown bandicoot) and *I. fusciventer* (quenda) across Australia with the number of samples used for each population represented by the size of the circle; and (**b**) translocation history of *I. auratus* (see maps (**a**) for an explanation of acronyms). Image of *I. auratus* sourced from Creazilla under an Attribution 4.0 International (CC BY 4.0) license. Image can be found at https://creazilla.com/nodes/64034-golden-bandicoot-clipart.

Australia recently released a national ten-year (2022–2032) plan for threatened species conservation and recovery, the “Threatened Species Action Plan: Towards Zero Extinctions”. The plan indicates that evidence-based conservation actions will be needed to achieve recovery targets. Genetic management will be integral to strengthening species’ resilience and adaptive capacity, as well as to ensure genetic diversity is maintained in insurance populations, particularly those in havens. In light of this ambition, here we conducted a genetic assessment of the threatened golden bandicoot *Isoodon auratus* (Ramsay 1887), a small (300–850 g) omnivorous marsupial. The remnant distribution of this species is heavily fragmented due to recent range collapse to near extinction on the mainland, attributed primarily to feral cat and fox predation and exacerbated by changed fire regimes and habitat loss (Woinarski et al. 2014, 2015). The naturally remnant populations of *I. auratus* are found on mainland Australia in the Kimberley and on offshore islands, including Barrow Island and Middle Island in the Pilbara region, Lachlan Island, Augustus Island, Storr Island and Uwins Island in the Kimberley, and Marchinbar Island in the Northern Territory (Woinarski et al. 2014) (Fig. 1a). Multiple single-source translocations of this species have occurred from Barrow Island to a fenced reserve (Matuwa Kurrara Kurrara National Park) (Lohr et al. 2021) and to Hermite Island and Doole Island in the Pilbara (Dunlop et al. 2021) (Fig. 1b). A captive population at Alice Springs Desert Park, Northern Territory, and a recently reintroduced population to Wild Deserts, New South Wales have been secondarily sourced from the Matuwa population. Populations have also been established on Guluwuru Island and Raragala Island using animals from Marchinbar Island. At least one more site is proposed for translocation: Newhaven in the Northern Territory (Fig. 1a).

The taxonomy of the species at both the specific and subspecific level has been in flux for several decades (summarised in Cooper et al. 2018; Jackson and Groves 2015; Thavornkanlapachai et al. 2021; Warburton and Travouillon 2016). For this study, we adhere to the delineation of three subspecies: *I. a. auratus* from the Kimberley region, *I. a. arnhemensis* from the Northern Territory and *I. a. barrowensis* from Barrow Island, Pilbara region (Jackson and Groves 2015). However, we note that *I. a. arnhemensis* (Lyne and Mort 1981) is variously recognised as a separate subspecies or merged with *I. a. auratus* in different treatments (Westerman et al. 2012), with only two subspecies, *I. a. barrowensis* (Thomas 1901) and *I. a. auratus* (merged with *I. a. arnhemensis*) listed independently as Vulnerable under the Environment Protection and Biodiversity Conservation (EPBC) Act 1999. Furthermore, recent molecular analysis suggests a pattern of intermediate polyphyly of *I. auratus* and *I. fusciventer* (Gray 1841) in mitochondrial DNA (Cooper et al. 2018; Thavornkanlapachai et al. 2021).

For *I. auratus*, decisions around population prioritisation and whether mixing within the three subspecies should be attempted are critical issues faced by conservation managers and organisations wishing to support the species’ recovery via reintroductions. Consequently, we aimed to (1) estimate the degree of genetic differentiation between each *I. auratus* sampling locality (hereafter referred to as population) and interpret it in an evolutionary context by comparing to other *Isoodon* taxa; (2) quantify the existing genetic variation within each population; (3) determine whether genetic diversity has been conserved in translocated populations, and (4) prioritise populations for conservation based on genetic contributions to within-species diversity. We expected to find strong structure between subspecies- and species-level taxa. Within species, we expected island populations to show low genetic diversity, particularly when established via translocations, and to be strongly differentiated from higher diversity mainland populations. Given the close relationship of *I. fusciventer* and *I. auratus*, we include samples from this species as well as *I. macrourus* (Gould 1842) (outgroup) to provide evolutionary context to patterns detected within *I. auratus* and to assist in establishing the relative scale of genetic differentiation between *I. auratus* subspecies, especially as strong structure is expected due to island isolation.

## Methods

### Sample collection, DNA sequencing, read assembly and filtering

Tissue samples were collected from 245 individuals and included 222 *I. auratus*, 9 *I. fusiventer*, and 14 *I. macrourus* samples (Fig. 1a, Table S1, and Supplementary Text).

DNA extraction procedures are detailed in the Supplementary Text. Library and ddRAD sequencing were performed at the Australian Genome Research Facility (AGRF) in Melbourne, Victoria. Illumina libraries were built on 280–375-bp DNA fragments and sequenced on the Illumina NextSeq 600 system (Supplementary Text).

Sequenced reads were cleaned, demultiplexed and trimmed to 125-bp with a phred quality score ≥30 using process_radtags module in Stacks v2.59 (Catchen et al. 2013). Samples with less than 400,000 reads (*n* = 28) were discarded. Parameters for the Stacks de novo pipeline were chosen on a subset of 100 random samples following the r80 optimisation approach of Paris et al. (2017), detailed in Supplementary Text. Two datasets were obtained through the de novo pipeline; the first including natural populations of *I. auratus*, *I. fusciventer* and *I. macrourus* (hereafter, the ‘*Isoodon*’ dataset, *n* = 134) and the second including all natural and translocated populations of *I. auratus* (hereafter, ‘*auratus*’ dataset, *n* = 222). Each dataset was run through the Populations module in Stacks with the following parameters: loci needed to be present in a single population, 50% of samples were required to process a locus, a maximum observed heterozygosity of 70%, and pruning to only a single SNP per locus to account for short distance linkage disequilibrium. The resulting VCF was filtered in R v4.0.2 using a modified script from Wright et al. (2019) and von Takach et al. (2020) with average allelic depth >2.5×, only retaining loci with a coverage difference between the reference and SNP allele <80%, iteratively filtering samples and SNPs with increasing thresholds reaching a final call rate of 90% in individuals and 95% in SNPs, minor allele count > = 3, and removing any loci with <100% reproducibility between technical replicates (see Fig. S1 for summary). Closely related individuals were estimated using the *beta.dosage* function in the *hierfstat* R package with pairwise kinship values > 0.25 considered closely related and removed (*n* = 7). Additional details on read assembly and data filtering methods are described in the Supplementary Text. Note that datasets were filtered in different ways to meet assumptions of analysis methods, as detailed in Fig. S1.

### Investigating population structure

Due to the apparent complex evolutionary histories of *Isoodon* species and the uncertainty in the taxonomic status (Cooper et al. 2018; Thavornkanlapachai et al. 2021), we investigated population structure using both the ‘*Isoodon*’ and ‘*auratus*’ datasets. We inferred population structure without prior knowledge of demographic history via principal coordinate analyses (PCoA) and Discriminant Analysis of Principle Components (DAPC). We used the *gl.pcoa* function in the *dartR* package (Gruber et al. 2018) to run the PCoA and the *find.clusters* function in the *adegenet* R package to run the DAPC (Jombart 2008). For DAPC, the number of components (PCs) was initially set to allow 90% of cumulative variance to be retained (50 PCs for ‘*Isoodon*’ dataset and 100 PCs for ‘*auratus*’ dataset) and then optimised using cross validation to choose the optimal number of PCs to retain (10 PCs and 30 PCs for the ‘*Isoodon*’ and ‘*auratus*’ datasets respectively). Successive DAPC analyses were run from K values 2–10 and the K value selected based on the lowest Bayesian information criterion (BIC) and after the largest decrease in BIC (Jombart et al. 2010). For the ‘*auratus*’ dataset, we also present analyses when K equalled the number of sampling locations (K = 15).

We estimated the proportion of an individual’s genome belonging to ancestral K gene pools using the sNMF function in the R package LEA (Frichot et al. 2014; Frichot and François 2015). We ranged the number of clusters from 1–10 with 1000 iterations and 100 repetitions, assessing the most likely K based on cross-validation and the entropy criterion (an informatic theoretic measure which reflects the number of ancestral populations that best explains the genotypic data). Since unequal population sizes can influence the estimation of ancestry proportions, we also re-ran the model subsampling each population to have ≤10 individuals (ranging from 1–10).

We further explored the evolutionary relationships among populations of the ‘*Isoodon*’ dataset as a maximum likelihood bifurcating tree using TreeMix version 1.13 (Pickrell and Pritchard 2012). This approach draws inferences from covariance in allele frequencies among populations by reconstructing population histories and whether populations represent independent divergence events. We first ran TreeMix 10 times for varying number of migration events (m) ranging from 0–10 (-global -k 500 -noss) using *I. macrourus* as an outgroup to root the tree. The optimal number of migration events was selected using the OptM R package (Fitak 2021) by estimating change points from threshold models (Sonderegger et al. 2009) and the variance explained by each migration event where the threshold of 99.8% of the variance explained is recommended (Pickrell and Pritchard 2012). We then ran TreeMix 50 times for each chosen migration event (m = 0 as null model, m = 1 and m = 6) and obtained a consensus tree and bootstrap values using the BITE R package (Milanesi et al. 2017). The residual covariance matrix was also estimated for each chosen migration event and the consensus tree using TreeMix.

We quantified the degree of genetic differentiation (F_ST_) and genetic divergence (D_xy_) between populations using the software pixy (Korunes and Samuk 2021). The ‘*Isoodon*’ and ‘*auratus*’ datasets were reparsed through the Populations module of Stacks (-p = 1, -r = 0.5) to produce a VCF consisting of both variant and invariant sites (--vcf-all) which was further filtered in VCFtools version 0.1.16 (Danecek et al. 2011) allowing 10% missing data (--max-missing = 0.9) and mean depth of loci between five and 100 (--min-meanDP = 5, --max-meanDP = 100). Pairwise summary statistics were computed in 10 kb windows. Populations with small sample size (*n* < 6) were excluded.

Using the ‘*auratus*’ dataset, including translocated populations, we tested for an isolation by distance model of differentiation by plotting geographic distance against F_ST_/(1-F_ST_) (Rousset 1997) and performed a Mantel test using the function *gl.ibd* in the dartR package.

### Assessing genetic variation of *Isoodon auratus*

To assess genetic diversity within populations, we first calculated the mean values of standard diversity parameters for each population with a sample size >6 using the ‘*auratus*’ dataset. Allelic richness (A_*R*_) was estimated using the *allelic.richness* functions in the *hierfstat* R package and the number of alleles which only occur in a single population (P_A_) using the *private_alleles* function in the *poppr* R package (Kamvar et al. 2014). Both metrics were standardised for unequal sample size. We followed the recommendations in Schmidt et al. (2021) when estimating heterozygosity to reduce biases associated with filtering, sample sizes and differences in allele frequencies among populations. This was achieved by re-calling variants in each population independently using individuals with less than 10% missing data (i.e. 90% genotyped) and 100% of individuals across all populations required to process a locus (-R = 100), thereby allowing no missing data across loci. Observed heterozygosity (H_O_), expected heterozygosity (H_E_) and nucleotide diversity (π) values were then extracted from Populations in Stacks summary statistics output. These independent datasets were also used to identify temporal changes in our translocated populations by calculating diversity metrics as described above across time periods. Due to only a single sample available in 2016 from Raragala Island, this population was excluded from this analysis. Autosomal heterozygosity estimates were calculated for the Guluwuru Island population as this method is more robust to small sample size (Schmidt et al. 2021), however, A_R_ was not calculated as sample sizes were <6.

### Prioritising populations of *Isoodon auratus* for genetic conservation

Using the ‘*auratus*’ dataset we investigated how genetic diversity was partitioned among the currently recognised subspecies as well as within and between populations using the *QDiver* function (Smouse et al. 2017) in GenAlEx v6.51b2 (Peakall and Smouse 2006, 2012). This method computes standardised genetic diversity metrics partitioned into hierarchical strata, and allows for evaluation of homo-/heterogeneity of within-stratum diversity components using a ‘Q’ metric derived from Rao’s Quadratic Entropy. The *QDiver* function creates a ‘diversity cascade’ that includes the total diversity of the species (γ), the diversity among subspecies (δ), the within-subspecies diversity (σ), the among-population diversity within each subspecies (β), and the within-population diversity within each subspecies (α). As this function does not allow for missing data, a dataset with a final call rate of 100% in SNPs was generated using the custom R script described above.

Secondly, we used the program Metapop2 (López-Cortegano et al. 2019) to assess the relative contribution of specific populations to both gene and allelic diversity across all populations sampled. This approach provides a statistically robust way to identify which populations contribute most to the overall genetic diversity of the species based on both local variation and genetic differentiation. The contribution of each population was estimated by removing that population and re-calculating the changes in within-population diversity (A_S_, H_S_), among-population diversity (D_A_, D_G_) and total allelic (A_T_) and gene (H_T_) diversity (López-Cortegano et al. 2019). In this instance, total allelic diversity is not the total number of alleles but is calculated as the average number of different alleles available in each pairwise grouping of populations (López-Cortegano et al. 2019). A positive value for a population means that on average, more genetic diversity is lost in the metapopulation when that population is removed while a negative value for a population indicates a gain in genetic diversity in the metapopulation when that population is removed. Using the Metapop2 program, we also simulated the optimum contribution of each population to a synthetic pool of 1000 individuals (e.g. a hypothetical translocation) to maximise heterozygosity (H) or the number of alleles (k). To account for unequal sample size between populations, we randomly subsampled each population (*n* = 6) and repeated the analysis 50 times, taking the average result across all runs.

Thirdly, we used the systematic planning approach of MARXAN (Watts et al. 2009) to identify combinations of extant populations that would best represent genetic diversity in the species (von Takach et al. 2021). In the absence of specific costed conservation options for each population, we allocated an equal unit cost of one to conserve each population and identified the optimal combination of populations to maximise allelic richness in the species, identifying optimal solutions for scenarios of 1–11 ‘protected’ populations using the R package ‘prioritizr’ (Hanson et al. 2022) and the SYMPHONY integer linear programming solver (Ralphs and Güzelsoy 2005). Using this method, each allele is considered a feature to be conserved, and each population is considered a planning unit. For each of 100 iterations, we randomly sampled six individuals per population, calculated the total number of alleles across all combined populations and identified a conservation solution for a maximum coverage (of alleles) objective for scenarios of 1–11. We tallied the number of configurations across the 100 replicates, as well as the resulting total allele count for each solution.

## Results

### Bioinformatic pipeline and filtering

Using the systematic evaluation of Stack parameters, both the number of polymorphic loci and number of SNPs increased with increasing *M* and *n* values; however, this increase reached an asymptote at *M* = *n* = 4 (Fig. S2) and therefore we chose this value for our de novo assembly. We ensured that coverage thresholds were on average greater than 25x to increase the robustness of our dataset to variation in sequence quality. Iterating m values resulted in mean coverage thresholds that ranged from 40.3× to 51.5× and therefore we deemed m = 3 to be suitable for our de novo assembly (Fig. S3).

Running the 134 *Isoodon* samples through the de novo pipeline (m = 3, M = 4, *n* = 4), our ‘*Isoodon*’ dataset retained 124,079 SNPs, with a mean read depth of 32.491 and 17.444 and a median read depth of 14.182 and 8.091 for the reference and alternative allele, respectively. After filtering using the custom R script, 117 unique individuals were retained with 8552 SNPs. When all missing data were removed for the TreeMix analysis, 117 individuals and 3043 SNPs were retained. Running 203 *I. auratus* samples that passed quality control (19 samples failed) through the de novo pipeline, 121,863 SNPs were retained, with a mean read depth of 26.088 and 11.436 and a median read depth of 11.282 and 5.222 for the reference and alternate allele, respectively. After filtering using the custom R script, 176 unique individuals and 8244 SNPs were retained, with an overall level of missing data for the filtered SNP by sample matrix of 1.3%. When all missing data were removed for the QDiver analysis, 172 unique individuals and 2495 SNPs were retained.

### Hierarchical population structure

**Fig. 2:**
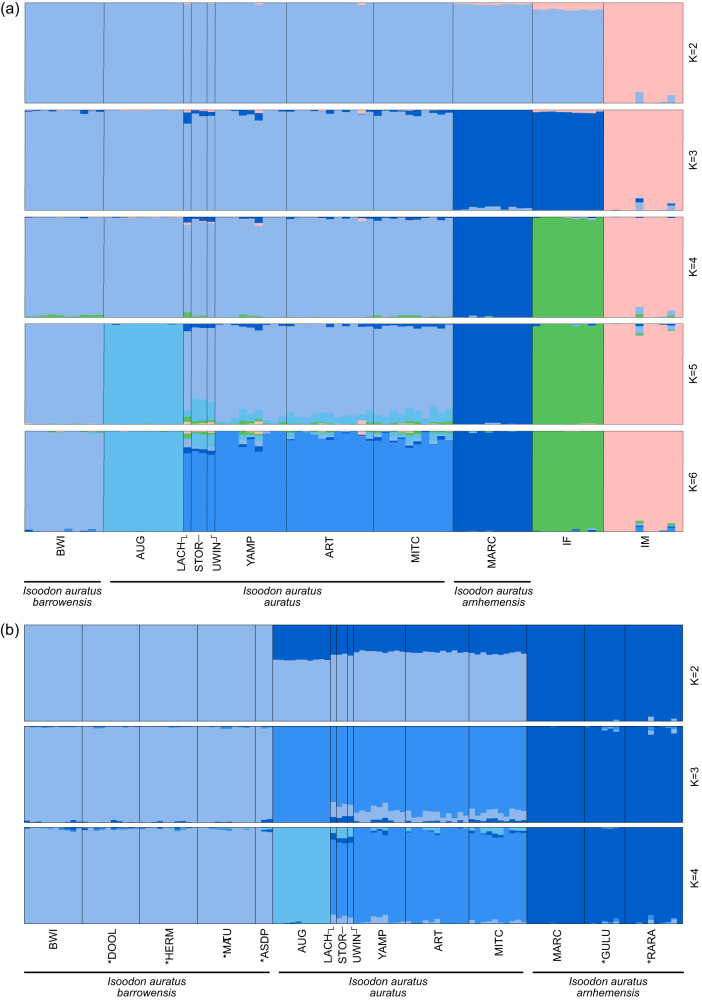
Patterns of hierarchical structuring amongst *Isoodon* populations are generally consistent with currently recognised species and subspecific nomenclature. Estimates of admixture coefficients for an individual in each population using the ‘sNMF’ function in the R package LEA at different K values when using (**a**) the ‘*Isoodon*’ dataset and (**b**) the ‘*auratus*’ dataset, where each population was subset to contain a maximum of 10 individuals. Populations include Barrow Island (BWI), Doole Island (DOOL), Hermite Island (HERM), Matuwa (MATU), Alice Springs Desert Park (ASDP), Augustus Island (AUG), Lachlan Island (LACH), Storr Island (STOR), Uwins Island (UWIN), Yampi Sound (YAMP), Artesian Range (ART), Mitchell Plateau (MITC), Marchinbar Island (MARC), Guluwuru Island (GULU), Raragala Island (RARA), *I. macrourus* (IM) and *I. fusciventer* (IF). Translocated populations are indicated by an asterisk (*).

Strong structure was observed between currently recognised subspecies and species nomenclature for both the ‘*Isoodon*’ and ‘*auratus*’ dataset in ‘sNMF’ analysis (Figs. 2a, b; S5), DAPC analysis (Fig. S6) and PCoA (Fig. S7). sNMF and DAPC analyses both resolved K = 6 and K = 4 for the ‘*Isoodon*’ and ‘*auratus*’ datasets respectively (Figs. S8, S9), although additional K values also revealed meaningful hierarchical population structuring (Fig. 2). There was some complexity in the way genetic relationships were visualised across analyses as would be expected with hierarchically structured populations. *Isoodon macrourus* was clearly distinct from *I. auratus* and *I. fusciventer* in sNMF and PCoA, and *I. fusciventer* showed a closer genetic relationship with *I. auratus*, as expected from their established taxonomic relationships. The relative placement of these species differed in DAPC analysis with the primary axis discriminating *I. auratus* and *I. fusciventer* (Fig. S6a) and minimising variation with *I. auratus and I. macrourus*. Island populations and Kimberley mainland populations (Artesian Range, Mitchell Plateau and Yampi Sound) from the ‘*auratus*’ dataset were consistently separated in all analyses, with the first two PC axes explaining 49% for the PCoA (Fig. S7) and 86% variation for the DAPC (Fig. S6b). Genetic differentiation of Marchinbar Island from remaining *I. auratus* was detected in sNMF and PCoA, yet minimised in DAPC. Augustus Island was consistently separated from the other populations, forming its own genetic cluster. Kimberley mainland populations clustered together and translocated populations consistently grouped with their source populations in the ‘sNMF’, PCoA and DAPC analyses (Figs. 2; S6c; S7).

**Fig. 3:**
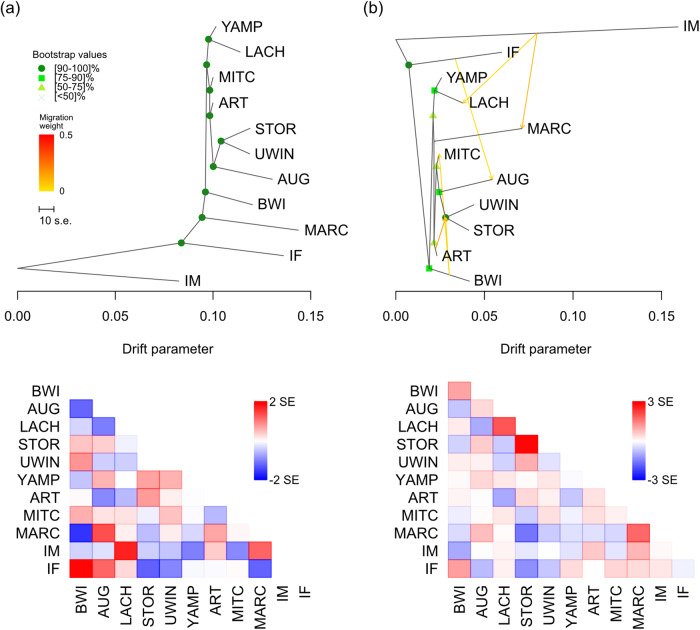
TreeMix consensus tree and bootstrap values displaying the relationships among populations as a bifurcating maximum-likelihood tree. (**a**) no migration edges (m = 0) and (**b**) six migration edges (m = 6), inferred as the best topology. Branch lengths on the horizontal axis represent the amount of genetic drift that has occurred along each branch. Bootstrap supports for each node are indicated. Below each tree is the associated residual fit of the observed versus the predicted squared allele frequency difference, expressed as the number of SE of the deviation. SE values are represented by colours according to the palette on the right. Residuals above zero indicate populations that are more closely related to each other in the data than in the best-fit tree and have potentially undergone admixture. Negative residuals represent populations that are less closely related in the data than represented in the best-fit tree. Populations include Barrow Island (BWI), Augustus Island (AUG), Lachlan Island (LACH), Storr Island (STOR), Uwins Island (UWIN), Yampi Sound (YAMP), Artesian Range (ART), Mitchell Plateau (MITC), Marchinbar Island (MARC), *I. macrourus* (IM) and *I. fusciventer* (IF).

Topology inferred from the TreeMix maximum likelihood tree with no migration events (Fig. 3a) was concordant with population clustering (Fig. 2). Six migration events were chosen to be the optimal value, explaining 99.77% of variation with a possible suboptimal number of a single migration event (Fig. S10). However, one migration event only explained 99.14% of variation (Fig. S10b). Tree topology did not change significantly with the addition of subsequent migration events (Figs. 3b, S11) and migration events had low weights (likely reflecting historic gene flow). Branch lengths supported strong drift in allele frequencies in the island populations, in contrast to the shorter branch lengths in the Kimberley mainland populations (Figs. 3, S11). Residuals from all models (m = 0, m = 1, m = 6; Figs. 3, S11b) revealed populations which may not fit a strict tree model where strongly positive residuals among some pairs indicated that these may be more closely related than they appear in the consensus tree and are potential candidates for admixture events.

Genetic differentiation (pairwise F_ST_) and nucleotide divergence (D_xy_) were variable between different named taxa as well as allopatric populations within taxa, as expected (Fig. S12). Translocated populations were not differentiated from their source population or other populations established from the same source (F_ST_ < 0.03, D_xy_ < 0.017), consistent with their clustering in the ‘sNMF’ analysis (Fig. 2b). For both the ‘*Isoodon*’ and ‘*auratus*’ datasets, pairwise genetic differentiation was greatest between Marchinbar Island and Augustus Island (F_ST_ = 0.65 and 0.57, D_xy_ = 0.112 and 0.125 for each dataset respectively). These islands also had the longest branch lengths in the TreeMix tree (Fig. 3). *Isoodon macrourus* had highest nucleotide divergence (D_xy_ = 0.159–0.168; Fig. S12a) followed by *I. fusciventer* (D_xy_ = 0.113–0.122). *Isoodon a. arnhemensis* had higher pairwise F_ST_ values (F_ST_ = 0.39–0.65) than *I. fusciventer* (F_ST_ = 0.2–0.54) and *I. macrourus* (F_ST_ = 0.33–0.57). Pairwise F_ST_ values of Augustus Island were higher than the other Kimberley mainland populations, consistent with clustering analyses (Figs. 2, S6, S7), however this was not reflected in the nucleotide divergence estimates which were very similar between each *I. a. auratus* population (Fig. S12). Increasing genetic differentiation among *I. auratus* populations was correlated with increasing geographic distance as supported by the mantel test for the isolation by distance analysis (r = 0.48, *p* = 0.003, 999 permutations; Fig. S13).

### Genetic diversity within populations

Mainland *I. auratus* populations had the highest genetic diversity (encompassing allelic richness, observed heterozygosity, expected heterozygosity and nucleotide diversity) as well as the highest number of private alleles, whilst the lowest diversity was observed in *I. a. arnhemensis* populations, which also had the lowest number of private alleles (Table 1, Fig. S14). Of all the natural island populations, Barrow Island had the highest genetic diversity, yet Augustus Island had the most private alleles (Table 1). Diversity metrics were similar between the two sampling time points in Doole Island, Hermite Island and Matuwa (Table S2), but were higher than Barrow Island (although note the difference in total number of sites called; Table 1). An increase was observed in heterozygosity and nucleotide diversity in the Guluwuru Island translocated population three years after this population was established (2009– 2011), despite low statistical power (Table S2).

**Table 1:** Population-level diversity metrics amongst *Isoodon auratus* populations: the number of samples (n), private alleles (P_A_), allelic richness (A_R_), autosomal H_O_, autosomal H_E_, nucleotide diversity (π) and the number of variant sites when each population is called independently.

		n	P_A_	A_R_	H_O_ (×100)	H_E_ (×100)	π(×100)	Variant sites(Total sites)
*Isoodon auratus barrowensis*	Barrow Island	43	125 (0.97)	1.16 (0.003)	0.16 (0.001)	0.16 (0.001)	0.17 (0.001)	22,400 (3,548,423)
	Doole Island	13	125 (1.1)	1.16 (0.003)	0.21 (0.001)	0.22 (0.001)	0.23 (0.001)	38,089 (5,332,658)
	Hermite Island	12	122 (1.12)	1.16 (0.003)	0.21 (0.001)	0.22 (0.001)	0.22 (0.001)	37,958 (5,386,248)
	Matuwa	16	132 (1.2)	1.16 (0.003)	0.22 (0.001)	0.23 (0.001)	0.23 (0.001)	30,110 (4,080,044)
*Isoodon auratus auratus*	Augustus Island	15	645 (1.13)	1.08 (0.002)	0.10 (0.001)	0.10 (0.001)	0.10 (0.001)	16,962 (5,022,314)
	Yampi Sound	6	951 (2.74)	1.23 (0.003)	0.29 (0.002)	0.34 (0.002)	0.37 (0.002)	53,218 (4,474,881)
	Artesian Range	9	913 (5.81)	1.23 (0.003)	0.31 (0.001)	0.35 (0.001)	0.37 (0.001)	85,194 (5,799,533)
	Mitchell Plateau	10	989 (4.77)	1.23 (0.003)	0.28 (0.001)	0.33 (0.001)	0.35 (0.002)	67,490 (4,712,664)
*Isoodon auratus arnhemensis*	Marchinbar Island	19	21 (0.81)	1.02 (0.001)	0.03 (0.001)	0.03 (0.00)	0.03 (0.00)	10,646 (4,988,195)
	Guluwuru Island	7	24 (0.39)	1.02 (0.001)	0.05 (0.001)	0.05 (0.00)	0.05 (0.001)	12,052 (5,554,659)
	Raragala Island	14	34 (0.84)	1.02 (0.001)	0.05 (0.001)	0.05 (0.00)	0.05 (0.001)	16,446 (4,500,567)

Standard errors are shown in parentheses. Note that P_A_ and A_R_ are standardised for sample size.

### Populations of high priority for genetic conservation

Partitioning genetic diversity into hierarchical strata via a standardised ‘Q’ diversity metric using the QDiver analysis revealed the unequal distribution of genetic diversity across the three recognised subspecies of *I. auratus* (Table 2; among ‘regions’ *δ* = 0.014—less than 15% of the grand total γ = 0.102). *Isoodon a. auratus* had statistically higher within-region diversity (σ = 0.123) and among-population diversity (β = 0.023) than the other subspecies restricted to offshore islands (*I. a. barrowensis* σ = 0.001 and β = 0.001; *I. a. arnhemensis* σ = 0.04 and β = 0; Bartlett’s test for homogeneity *p* = 0.001). Within populations, Artesian Range (α = 0.128) had slightly more diversity than Mitchell Plateau (α = 0.124), which was not detected in diversity metrics (Table 1). Augustus Island had a significantly lower level of diversity than the other *I. auratus* mainland populations (α = 0.082 in comparison to α = 0.117, α = 0.128, α = 0.124 for Yampi Peninsula, Artesian Range and Mitchell Plateau, respectively; Bartlett’s test for homogenous *p* = 0.047).

**Table 2:** Diversity cascade for *Isoodon auratus*, showing the hierarchical stratification of genetic diversity held within and among the three recognised subspecies.

Parameter	Subspecies	Population	Diversity value	*p* value (Bartlett’s test)
γ = Q(GT)			0.102	
*δ* = Q(AR)			0.014	
σ = Q(WR)	*I. a. barrowensis*		0.091	0.001
	*I. a. auratus*		0.123	
	*I. a. arnhemensis*		0.040	
β = Q(AP/WR)	*I. a. barrowensis*		0.001	0.001
	*I. a. auratus*		0.023	
	*I. a. arnhemensis*		0.000	
α = Q(WP/WR)	*I. a. barrowensis*	Barrow Island	0.090	0.979
		Doole Island	0.091	
		Hermite Island	0.089	
		Matuwa	0.089	
	*I. a. auratus*	Augustus Island	0.082	0.089
		Yampi Peninsula	0.117	
		Artesian Range	0.128	
		Mitchell Plateau	0.124	
	*I. a. arnhemensis*	Marchinbar Island	0.039	0.260
		Guluwuru Island	0.039	
		Raragala Island	0.040	

*GT* grand total, *AR* among subspecies, *WP/WR* within population within subspecies.

**Fig. 4:**
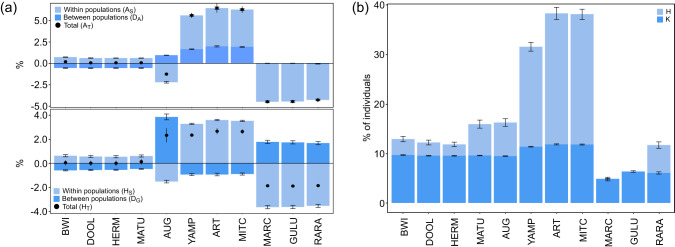
The relative contributions of *Isoodon auratus* populations to the genetic diversity of the species. Contributions to (**a**) within- (A_S_, H_S_; light blue), between (D_A_, D_G_; dark blue) and total (A_T_, H_T_; black dot) allelic and gene diversity, respectively, of *I. auratus* populations: Barrow Island (BWI), Doole Island (DOOL), Hermite Island (HERM), Matuwa (MATU), Augustus Island (AUG), Yampi Sound (YAMP), Artesian Range (ART), Mitchell Plateau (MITC), Marchinbar Island (MARC), Guluwuru Island (GULU), Raragala Island (RARA); and (**b**) percentage of individuals from each population to a synthetic pool of individuals to maximise gene diversity (H) and/or number of alleles (k).

The rank of contributions (from the MetaPop2 analysis) to total diversity of the species was similar for gene and allelic diversity, except for Augustus Island which had a negative contribution to allelic (A_T_) diversity but a positive contribution to gene (H_T_) diversity (Fig. 4a). As A_T_ reflects the number and distribution of alleles across populations (López-Cortegano et al. 2019), this decrease likely reflects the private alleles present in Augustus Island (Table 1) that would be lost if this population is removed. There were profound differences between these two diversity components across populations, with mainland *I. a. auratus* populations having the greatest contribution to within-population allelic (A_S_) and gene (H_S_) diversity (Fig. 4a), consistent with within-region diversity of the QDiver analysis (Table 2). These populations, as well as Augustus Island, also had the greatest contribution to between-population allelic diversity (D_A_) which coincided with these populations having the greatest number of private alleles (Table 1). Populations of *I. a. arnhemensis* had a substantial negative contribution to within-population diversity (A_S_, H_S_), consistent with low estimates of genetic diversity metrics (Table 1) and no among-population diversity in the QDiver analysis (Table 2). Conversely, these populations, as well as Augustus Island, contributed most significantly to between-population gene (D_G_) diversity, which reflected their higher pairwise F_ST_ values (Fig. S12). When the optimal contribution (%) of individuals to a synthetic pool was computed, the contribution to the number of alleles (k) was similar between populations but *I. a. auratus* mainland populations had the largest contribution to heterozygosity (H) (Fig. 4b).

**Fig. 5:**
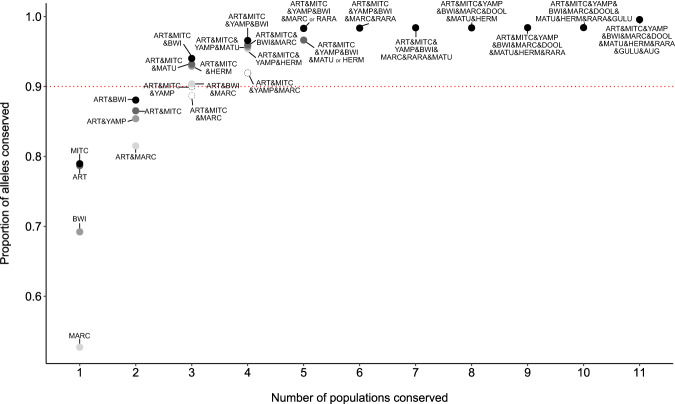
Impacts of conserving differing numbers of populations for *Isoodon auratus* on the proportion of total alleles conserved across the species where the red dotted line indicates 90% of alleles are conserved. Populations include Barrow Island (BWI), Doole Island (DOOL), Hermite Island (HERM), Matuwa (MATU), Alice Springs Desert Park (ASDP), Augustus Island (AUG), Lachlan Island (LACH), Storr Island (STOR), Uwins Island (UWIN), Yampi Sound (YAMP), Artesian Range (ART), Mitchell Plateau (MITC), Marchinbar Island (MARC), Guluwuru Island (GULU), and Raragala Island (RARA).

Our MARXAN approach suggested that either the Artesian Range or Mitchell Plateau populations from the mainland were the most effective single populations to conserve allelic richness across the species, representing 79% of all alleles detected (67% and 33% of iterations selected these populations respectively; Fig. 5; Table S3). At least three populations were necessary to conserve 90% of alleles across the species (Fig. 5), with populations from *I. a. auratus* and *I. a. barrowensis* always being selected (Table S3). Populations from the Artesian Range, Mitchell Plateau and Barrow Island together retained 94% of alleles. Conserving five to ten of the *I. auratus* populations sampled in this study consistently retained 98% of alleles (Fig. 5), with populations from the Kimberley mainland always being selected (Table S3). Despite Augustus Island never being selected in an optimal scenario (Table S3), all 11 populations would need to be conserved to retain 100% of remaining genetic diversity of *I. auratus*, reflecting some unique diversity present within Augustus Island (Table 1).

## Discussion

Here we confirmed genetic distinction of *I. auratus* from *I. fusciventer* and found that the three subspecies of *I. auratus*, representing the remnant Kimberley mainland, Barrow Island and Marchinbar Island populations, showed strong genetic differentiation as anticipated due to their long-term geographic isolation. A fourth genetic cluster, Augustus Island in the Kimberley, adjacent to the only remnant mainland population, was also resolved within *I. auratus* that had not been previously recognised. Consistent with our expectations, all island populations had lower genetic diversity relative to the mainland populations, contributing to their genetic distinctiveness, as genetic differentiation metrics are influenced by the level of within-population variance. Hierarchical diversity analyses indicated that the remnant Kimberley mainland population of *I. auratus* represents the greatest reservoir of genetic diversity within the species, with secondary contribution from Barrow Island. As such, these populations are a high priority to safeguard against future decline and to ensure the preservation of >90% of remaining species-level diversity. Our findings show the importance of understanding the genetic relationships among populations, particularly remnant island populations, and the selection of appropriate combinations of source animals for reintroduction and ecosystem restoration programs to maximise genetic diversity and adaptive potential.

### Conserving genetic diversity in highly structured populations

As genetic diversity is not evenly distributed across populations, it is essential to consider patterns of population genetic structure when developing strategic frameworks for the management of threatened species to ensure that most genetic diversity within a species is conserved. Our prioritization analyses indicate that populations most important for conserving genetic diversity of *I. auratus* consisted primarily of *I. a. auratus* ancestry, highlighting the importance of the Kimberley mainland populations as a major reservoir of genetic diversity for this species. Furthermore, as diversity loss tends to be higher on islands (Leigh et al. 2019), well connected, large populations on the mainland may better support long-term retention of genetic diversity for this species. Currently, the spatial extent of these populations across the Kimberley region, and their population sizes, are unknown, although survey work is underway (Sayers et al. 2022).

Introduced predators, inappropriate fire regimes, and habitat degradation and fragmentation are known threats to populations of *I. auratus* (Woinarski et al. 2014) and numerous other Australian species in northern Australia (Carwardine et al. 2012; Geary et al. 2019; Kearney et al. 2019; von Takach et al. 2020). Yampi Sound is recognised as a ‘priority place’ in Australia’s national Threatened Species Action Plan, acknowledging a focus on threatened species which share the same habitat to support recovery of multiple species. As the majority of currently known *I. auratus* populations already exist within conservation land tenures and Indigenous Protected Areas, ongoing management in collaboration with Traditional Custodians to mitigate the impact of these threatening processes on the Australian mainland are a high priority (Carwardine et al. 2012; von Takach et al. 2020; von Takach et al. 2022a), with a particular emphasis on protecting critical habitat and maintaining its connectivity.

In the past, northern Australia has been considered to provide a level of conservation security for biodiversity, particularly for mammals, yet population declines are being observed (Davies et al. 2018; Woinarski et al. 2011). This region offers the only remaining refugia for *I. auratus* on the mainland, given the species’ disappearance from 95% of its extensive historic distribution (Woinarski et al. 2014) (Fig. 1a). The large geographic contraction and subsequent population declines have likely led to a massive loss in historical genetic diversity, potentially exceeding 33% (see Exposito-Alonso et al. 2022). This loss, in conjunction with compounding impacts of ongoing threats, may hinder the species’ ability to recolonize large areas of its fundamental niche (Woinarski et al. 2011). This pattern aligns with previous findings for Australian rodents, where extinct populations with large geographic distributions exhibited notably higher heterozygosity compared to extant species with small/restricted remnant populations, such as the Shark Bay mouse (*Pseudomys gouldii*) (Roycroft et al. 2021). Given the role that *I. auratus* Kimberley mainland populations play as a crucial repository of genetic diversity, it is imperative to safeguard these populations to ensure the long-term survival of the species.

In this study, island populations showed lower levels of genetic variability relative to mainland Kimberley populations, suggesting that islands have suffered a loss of genetic variation through genetic drift and population bottlenecks caused by their geographic isolation. This is particularly evident for *I. a. arnhemensis* populations in the Northern Territory that have substantially lower diversity relative to the other populations sampled and make a limited contribution to the overall diversity of the species (Table 1; Fig. 4). Similar results have been found for the Marchinbar Island population of the northern quoll (*Dasyurus hallucatus*), which displayed extremely low levels of genetic diversity compared to the mainland (von Takach et al. 2022b). Small populations on islands are susceptible to losing genetic diversity due to strong drift and are more likely to accumulate deleterious mutations (Lohr and Haag 2015; Weeks et al. 2016). However, populations with low genetic diversity are also known to have persisted for long periods of time (e.g. Westbury et al. 2019). Additional monitoring on these Northern Territory islands could assist in understanding whether the observed low diversity is indicative of demographic decline or may result in reduced population fitness.

While the relative importance of *I. a. arnhemensis* populations for the preservation of neutral genomic diversity as represented by these genomic SNPs at the species level is somewhat limited, the loss of these populations could result in the loss of a possible subspecies and the loss of ecological function on remnant islands. Furthermore, *I. auratus* has already undergone a large range contraction and possibly already suffered a loss in diversity exceeding the preservation targets of international guidelines. Conservation efforts should aim to retain all remaining genetic diversity of the species, which would entail the conservation of populations across all three subspecies, with a precedence in conserving the mainland populations.

### Incorporating genetic information into species’ management

To preserve the long-term viability, resilience, and adaptive potential of *I. auratus*, genetic mixing between islands or between mainland and islands is increasingly being considered (Hoffmann et al. 2021; Liddell et al. 2021). The option of genetic mixing, even between subspecies, could be a worthy endeavour to accentuate adaptive potential to keep pace with changing environments (Brauer et al. 2023; Chan et al. 2019; Zecherle et al. 2021). This has been shown to be successful in a range of species and advocated as a potentially valuable conservation tool (Brauer et al. 2023; Chan et al. 2019; Harrisson et al. 2016; Rick et al. 2019; Taylor and Larson 2019; Undin et al. 2021; Weeks et al. 2017). Given that the Kimberly mainland (Artesian Range and Mitchell Plateau) as well as Barrow Island were key to retaining >90% allelic diversity in the species (Fig. 5), we recommend that mixing these populations should be considered for future reintroductions. While there are potential risks when crossing highly diverged populations, namely outbreeding depression and genetic swamping (Edmands 2007; Frankham et al. 2011; Muhlfeld et al. 2009), these risks may be exaggerated (Frankham 2015; Liddell et al. 2021; Ralls et al. 2018; Weeks et al. 2016). At a minimum, future translocations of *I. auratus* into mainland reserves or feral predator-free islands should consider sourcing from the Kimberley mainland to ensure that the important genetic diversity from this region is preserved within the conservation ‘safe havens’ network, within which only a single population (Barrow Island) is currently represented.

Given the success of reintroductions of this species to date, translocations will continue to be an important conservation tool to reduce the risk of extinction for threatened species and can ensure persistence of important genetic variation (Weeks et al. 2011). Petroleum engineering companies operating on Barrow Island have funded multiple translocations of mammals to found insurance populations elsewhere, via environmental offsets linked to environmental approvals (Dunlop et al. 2021). Our analyses indicate genome-wide diversity of founder groups has been maintained (>98%) in the translocated populations on Hermite and Doole Islands and at Matuwa, exceeding recommendations in translocation guidelines (Frankham et al. 2014; Weeks et al. 2011) and expectations from populations viability analyses (Ottewell et al. 2014). This is likely the consequence of relatively large founder sizes (92–160 individuals; Fig. 1b), consistent with multiple studies highlighting the importance of establishing new populations with large numbers (*n* > 100; Ottewell et al. 2014; Weeks et al. 2011).

Nevertheless, ongoing monitoring is necessary to ensure that genetic diversity is conserved over time in translocated populations, as changes in population size, structure, and selection pressure can lead to genetic changes or erosion over the long-term. Periodic monitoring (recommended at least every five years based on PVA modelling, see Ottewell et al. 2014) will be required to ensure that genetic diversity within these populations persists, with more frequent monitoring recommended after catastrophic events (i.e. drought or disease) and when sourcing animals for translocations. While we attempted to investigate temporal changes in the Guluwuru Island, we were limited by small sample size between years. Further sampling of the populations translocated to Raragala and Guluwuru Islands would be valuable to assess the population trajectories. Regular monitoring of the Northern Territory populations may also be useful to ensure that the relatively low genetic diversity observed in these populations does not impact their health (e.g. declining population size due to inbreeding effects). Furthermore, periodic genetic monitoring across the Kimberley mainland would serve as a valuable metric, not only to ensure genetic diversity is maintained in these populations but also as a useful indicator of whether other management actions are successfully maintaining occupancy and connectivity across the Kimberley.

Australia’s heavy reliance on offshore islands for threatened species management, whilst crucial for the persistence of multiple mammal species (Legge et al. 2018), leads to many challenges when planning conservation efforts across a species more generally. Even when populations on islands are relatively large and stable, declines are still observed (Davies et al. 2018) and subsequently their persistence is questionable without the intervention of active conservation actions. Foremost of interest when considering genetic management, is that island populations are consistently deemed to be unique lineages despite insufficient evidence that any differentiation is adaptive (Wolf and Ellegren 2017). While island populations of *I. auratus* appear to be genetically distinct from one another and the mainland, genetic differentiation measures are often heavily influenced by changes in allele frequencies and within population variance (Weeks et al. 2016). Subsequently we observed populations with the lowest diversity metrics to be the most genetically ‘distinct’, namely Marchinbar Island. The population structure observed in our analyses support the premise that islands have experienced independent histories to such an extent that allele frequencies are clearly differentiated. However, the low genetic divergence (D_XY_) observed between *I. auratus* populations and long branch lengths in the TreeMix analysis indicated a strong influence of population-specific genetic drift. Other Kimberley Islands in closer proximity to the mainland (Lachlan Island, Storr Island and Uwins Island), although excluded from most analyses due to small sample size, were not distinguished in the clustering analyses. Therefore, divergence time of islands from the mainland and ecologically relevant phenotypic variation should also be considered. The identified hierarchical structure generally coincides with the three subspecies of *I. auratus*, but this structure primarily reflects geographic isolation and thus should not constrain management options by defining discrete units. In the case of reintroduction programs, prioritizing the conservation of populations for their genetic diversity (with the aim of maintaining >90% of remaining diversity), and thus their adaptive potential, should take precedence over preserving the perceived genetic uniqueness of island populations. This approach ensures the conservation of the species’ overall genetic variability, resilience, and adaptive capacity.

We still lack an understanding of how genomic differences translate directly into population dynamics in natural populations, especially when translocating them to novel environments (Seaborn et al. 2021). Genetic mixing and associated eco-evolutionary feedbacks can be unpredictable, thereby data-driven approaches are needed to guide best practice (Aisya et al. 2022; Frankham et al. 2011; Hoffmann et al. 2015; Rossetto et al. 2021; Seaborn et al. 2021). An adaptive management framework would therefore be appropriate in undertaking specific conservation actions, including mixing of divergent populations, and scientifically evaluating their outcomes to inform future conservation approaches. Our work provides an excellent model to encourage conservation managers to embrace the complexity of integrating multiple practices, including genomics, in decision-making frameworks for an adaptive management approach.

## Concluding remarks

While remnant island populations harbour a proportion of the genetic diversity present within *I. auratus*, this differentiation should be considered in the context of genetic processes. Genetic drift and within-population variance are likely driving the apparent differentiation among these geographically isolated islands. In this aspect, separate genetic management of populations may hinder the species’ ability to adapt to future environmental change and thus conservation goals for species recovery should be targeted at the species level. Our findings emphasize the critical contribution of mainland Kimberley populations in conserving the genetic diversity of *I. auratus*. Future translocations should aim to safeguard sufficient genetic diversity (>90%) at the species level, with insurance populations containing representatives of each lineage to spatially spread the risk of cumulative threats and catastrophes. Overall, our study highlights the importance of understanding population genetic structure when considering the loss of genetic diversity across genetically diverged and fragmented populations, particularly islands, and how such information is crucial to incorporate into conservation strategies and management of threatened species.

### Supplementary information

Supplementary material

## Data Availability

All raw sequencing data has been uploaded to the Oz Mammal Genomics Initiative data portal (https://data.bioplatforms.com/organization/about/bpa-omg). All bioinformatics and R scripts along with relevant metadata have been uploaded to the Mendeley Data Repository (https://data.mendeley.com/datasets/32kz25fzy9/1).
