# Supplementary material for: Population genomic diversity and structure in the golden bandicoot: a history of isolation, extirpation, and conservation
Source: Heredity (Edinb). 2023 Oct 8;131(5-6):374–86. doi: 10.1038/s41437-023-00653-2 (PMC10673901; doi:10.1038/s41437-023-00653-2)
Supplement: Supplementary file 1 — Supplementary material [file 41437_2023_653_MOESM1_ESM.docx]

Supplementary material

# **Population genomic diversity and structure in the golden bandicoot: a history of isolation, extirpation, and conservation**

Kate Rick, Margaret Byrne, Skye Cameron, Steve J.B. Cooper, Judy Dunlop, Brydie Hill, Cheryl Lohr, Nicola J. Mitchell, Craig Moritz, Kenny J. Travouillon, Brenton von Takach, Kym Ottewell

# Methods

## Sample collection, DNA sequencing, read assembly and filtering

We collated tissue samples (2 mm ear biopsies, stored in 70% ethanol) from multiple researchers and organisations (see Acknowledgements) who trapped *Isoodon* individuals during a range of fieldwork and monitoring programs. The total number of samples obtained for DNA extraction and subsequent double-digest restriction-site associated DNA (ddRAD) sequencing was 245, and included 222 *I. auratus*, 9 *I. fusciventer*, and 14 *I. macrourus* individuals. Samples of *I. auratus* covered most of the extant distribution of the species (Figure 1a), with sample sizes ranging from 1–25 per locality (Table S1).

DNA was extracted following a standard salting out extraction method (Sunnucks & Hales 1996) and quantified using a broad-range dsDNA assay kit for the Qubit Fluorometer 3.0, with samples normalised to 10 ng/μl in 25 μl (250 ng total) for library preparation. The 245 samples (plus 16 technical replicates) were sent to the Australian Genome Research Facility (AGRF) in Melbourne, Victoria, for ddRAD library preparation and sequencing. Briefly, 50 ng of gDNA for each sample was digested using the restriction enzyme combination of *Pst*I-*Msp*l and ligated with unique barcoded adapters compatible with the restriction site overhang. Samples were purified, with size selection (280-375 bp) carried out on a Blue Pippin (Sage Science). Libraries were PCR amplified with indexed primers and sequencing carried out on the Illumina NextSeq 600 with 150 cycles in HIGH-output mode over two flowcells.

Raw sequence reads were screened for quality using FastQC (Andrews 2010) and run through the Stacks v2.59 (Catchen et al. 2013) *de novo* pipeline for read processing, locus assembly and variant calling. Approximately, 1.38 billion raw sequenced reads were cleaned, demultiplexed and trimmed to 125 bp (-t 125) with a phred quality score ≥ 30 (-s 30) through the process_radtags module with 1.09 billion reads retained. Samples with fewer than 400,000 reads (n = 28) were discarded from downstream analyses. A combination of read assembly parameters within the *de novo* map pipeline were tested and optimised on a subset of 100 random samples, ensuring representation across all populations (including *I. fusciventer* and *I. macrourus)*, following the r80 optimisation approach of Paris et al. (2017). This method consists of systematically varying locus assembly parameters and monitoring the number of polymorphic loci found in 80% of samples or more, until a stable set of values for m (the minimum number of identical raw reads required to create a stack), M (the minimum number of mismatches allowed between loci when processing a single individual) and n (the number of mismatches allowed between stacks during construction of the catalogue) are found. First, m was set to three, as this value performs well for a broad range of datasets (Rochette & Catchen 2017), and M and n were varied from one to seven but kept equal (M=n). To ensure sufficient coverage, m was also varied from three to six while setting the default values of M = 2 and n = 1.

We obtained two datasets through the *de novo* pipeline using optimal parameters; the first including samples from naturally extant populations of *I. auratus*, *I. fusciventer* and *I. macrourus* (hereafter, the ‘*Isoodon*’ dataset, n=134) and the second using only samples of *I. auratus*, including naturally extant and translocated populations (hereafter, ‘*auratus*’ dataset, n=222). For each dataset, the Populations module in Stacks was run with the following parameters: loci needed to be present in a single population (-p=1), 50% of samples needed to be genotyped to process a locus to avoid bias that may result from calling SNPs from highly divergent populations (-r=0.5), a maximum observed heterozygosity of 70% to exclude erroneous merging of paralogous loci (--max_obs_het=0.7), and pruning to only a single SNP per locus to account for short distance linkage disequilibrium (--write_random_snp).

The resulting VCF file was further filtered in R v.4.0.2 using a modified script from Wright et al. (2019) and Von Takach et al. (2020) with average allelic depth >2.5x and only retaining loci with a coverage difference between the reference and SNP allele <80%. Samples and SNPs were then iteratively filtered with increasing thresholds reaching a final call rate of 90% in individuals and 95% in SNPs. Further putatively erroneous loci were removed by filtering on a Minor Allele Count (MAC) >=3 and removing any loci with <100% reproducibility between technical replicates. None of the retained loci were monomorphic after all filtering steps (see Figure S1 for summary).

To remove individuals which were closely related, we estimated pairwise kinships between individuals within a single population using the *beta.dosage* function in the *hierfstat* R package. As differences in allele frequencies among populations can lead to biases in population genetic parameter estimation, Schmidt et al. (2021) recommends analysing populations in independent runs when possible. Therefore, SNPs were re-called individually per population to calculate pairwise relatedness and each independent dataset was filtered as described above. Pairwise kinship values greater than 0.25 were considered closely related (n=7) and these individuals were removed for all downstream analyses (Figure S4).

# Results

Table S1. Summary of samples used in this study.

| Population | Taxa | Abbreviations used in this study | Island vs mainland | Remnant vs translocated | No. of samples sequenced | No. of samples post-filtering |
| --- | --- | --- | --- | --- | --- | --- |
| Barrow Island | *Isoodon auratus barrowensis* | BWI | Island | Remnant | 43 | 43 |
| Doole Island | *Isoodon auratus barrowensis* | DOOL | Island | Translocated | 13 | 13 |
| Hermite Island | *Isoodon auratus barrowensis* | HERM | Island | Translocated | 12 | 12 |
| Matuwa Kurrara Kurrara National Park | *Isoodon auratus barrowensis* | MATU | Mainland (fenced reserve) | Translocated | 124* | 16 |
| Alice Springs Desert Park | *Isoodon auratus barrowensis* | ASDP | Mainland (captive) | Translocated | 3 | 3 |
| Augustus Island | *Isoodon auratus auratus* | AUG | Island | Remnant | 17 | 15 |
| Lachlan Island | *Isoodon auratus auratus* | LACH | Island | Remnant | 1 | 1 |
| Storr Island | *Isoodon auratus auratus* | STOR | Island | Remnant | 2 | 2 |
| Uwins Island | *Isoodon auratus auratus* | UWIN | Island | Remnant | 1 | 1 |
| Yampi Sound | *Isoodon auratus auratus* | YAMP | Mainland | Remnant | 12 | 6 |
| Artesian Range | *Isoodon auratus auratus* | ART | Mainland | Remnant | 12 | 9 |
| Prince Reagent National Park / Mitchell Plateau | *Isoodon auratus auratus* | MITC | Mainland | Remnant | 12 | 10 |
| Marchinbar Island | *Isoodon auratus arnhemensis* | MARC | Island | Remnant | 24 | 19 |
| Guluwuru Island | *Isoodon auratus arnhemensis* | GULU | Island | Translocated | 9 | 7 |
| Raragala Island | *Isoodon auratus arnhemensis* | RARA | Island | Translocated | 16 | 14 |
| South-west Western Australia | *Isoodon fusciventer* | IF | Mainland | Remnant | 9 | 9 |
| Kimberley region, Western Australia | *Isoodon macrourus* | IM | Mainland | Remnant | 14 | 14 |

*Only 16 samples from 2019 (most recent sampling period) were included in this study


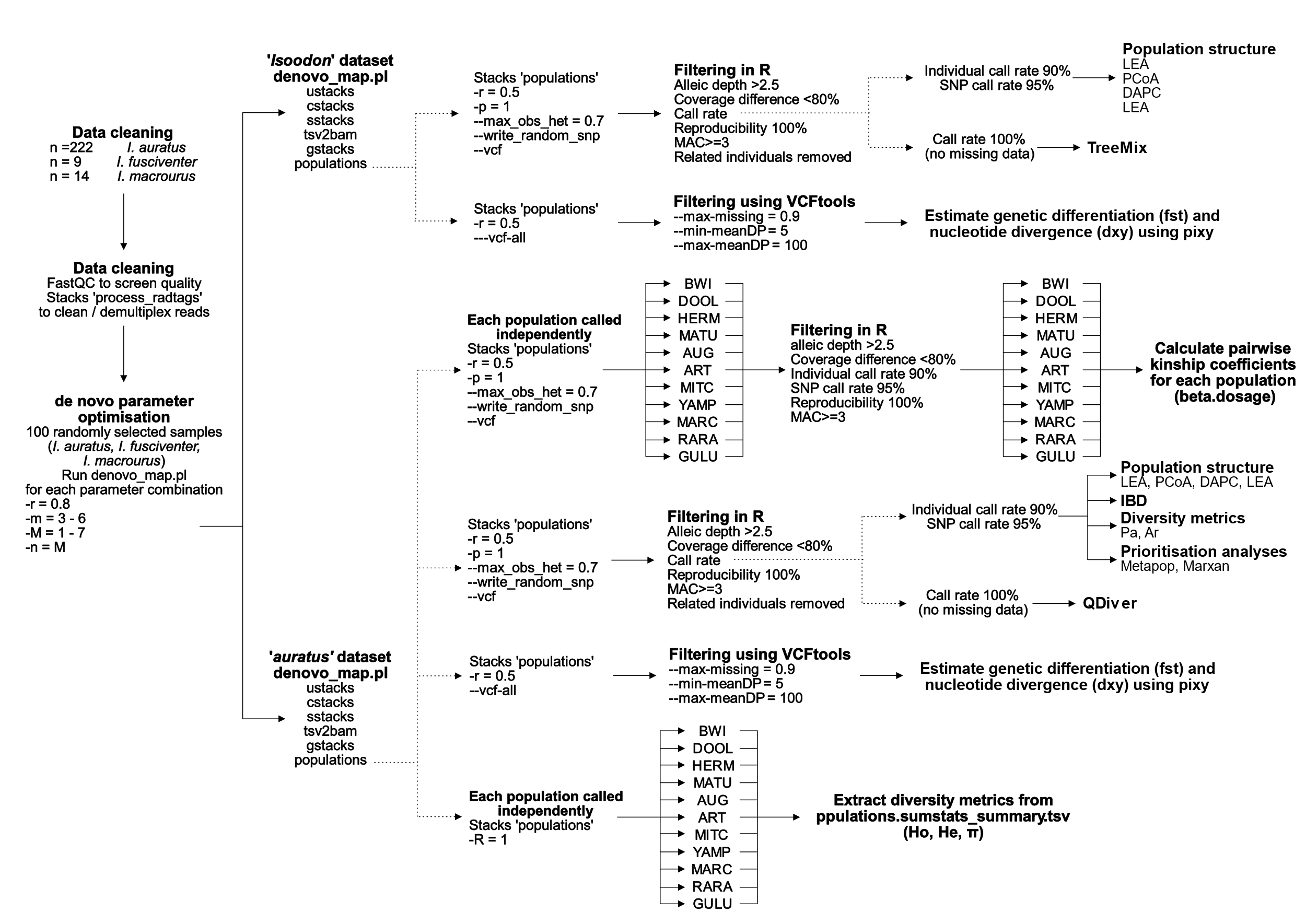


Figure S1. Overview of filtering used for each dataset and analysis.


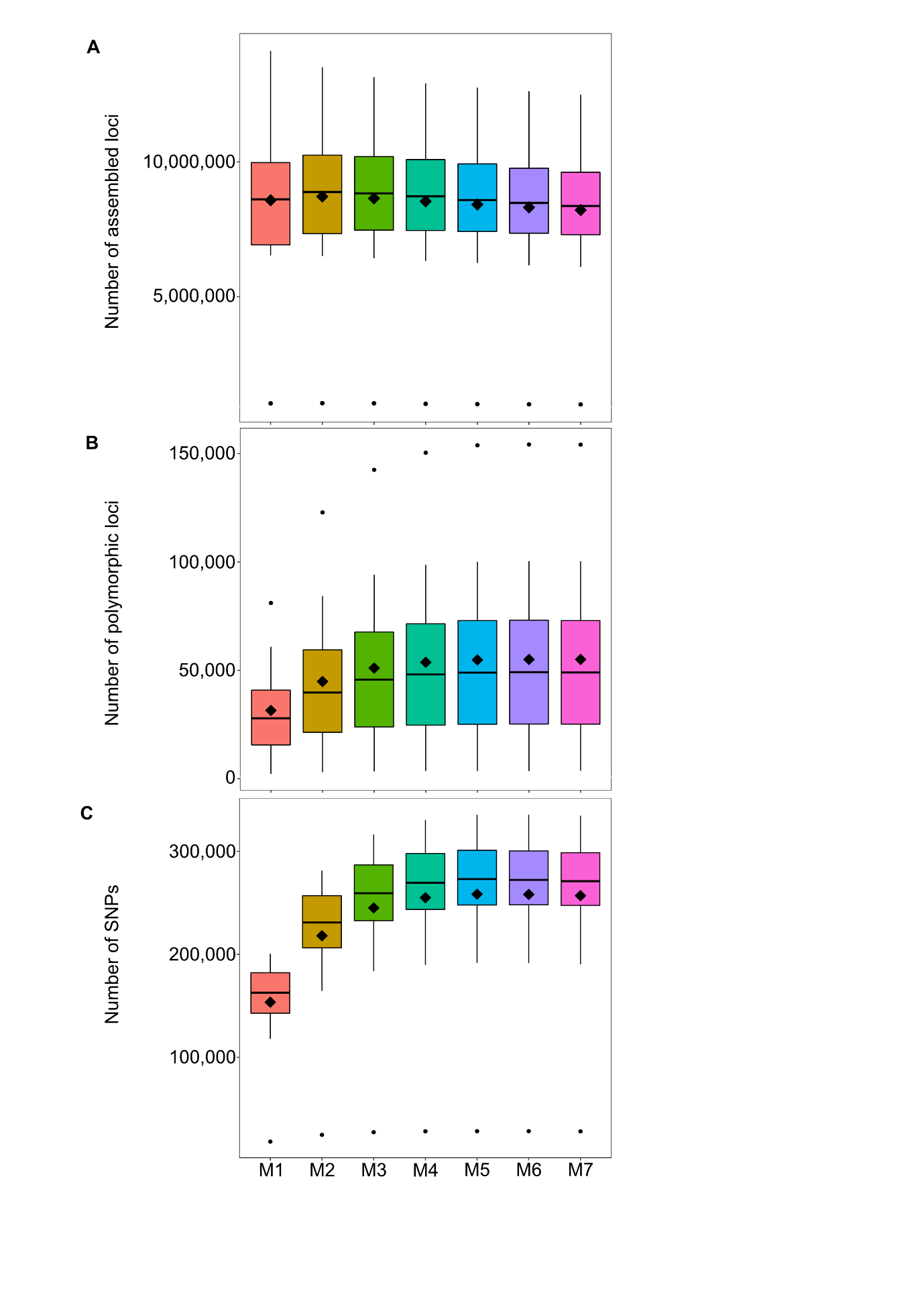


Figure S2. Boxplots of iterating values of the minimum number of mismatches allowed between loci when processing a single individual (M) to evaluate the effects of varying M parameters on (a) the number of assembled loci, (b) the number of polymorphic loci, and (c) the number of SNPs. Black diamond reflects the mean, middle horizontal line reflects the median, the boxes are bound by the 25^th^ and 75^th^ quartiles and the vertical lines show the minimum and maximum range of values.


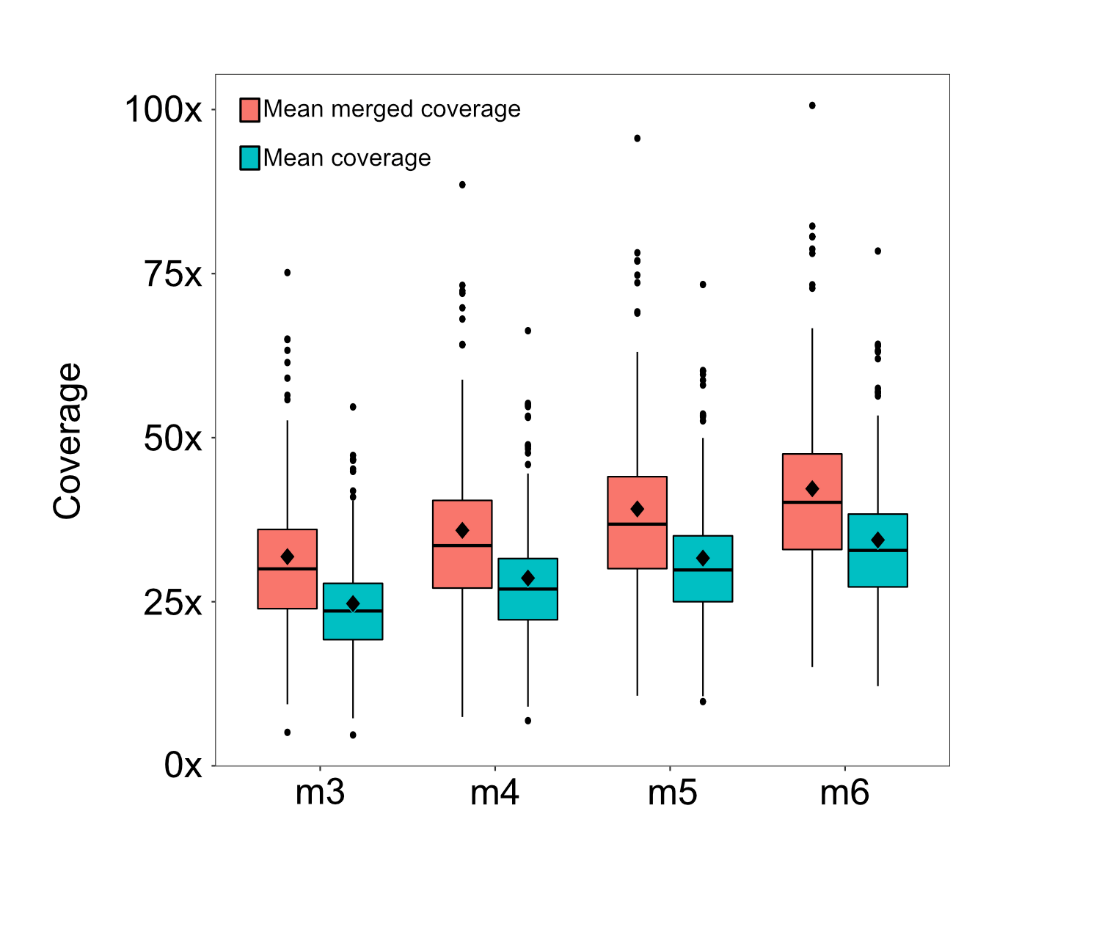


Figure S3. Boxplots depicting the sequencing coverage across individuals, where mean coverage (green) is the average coverage for primary reads and mean merged coverage (pink) is the coverage after merging alleles. Mean is indicated by a black diamond, middle horizontal line reflects the median, the boxes are bound by the 25^th^ and 75^th^ quartiles and the vertical lines show the minimum and maximum range of values.


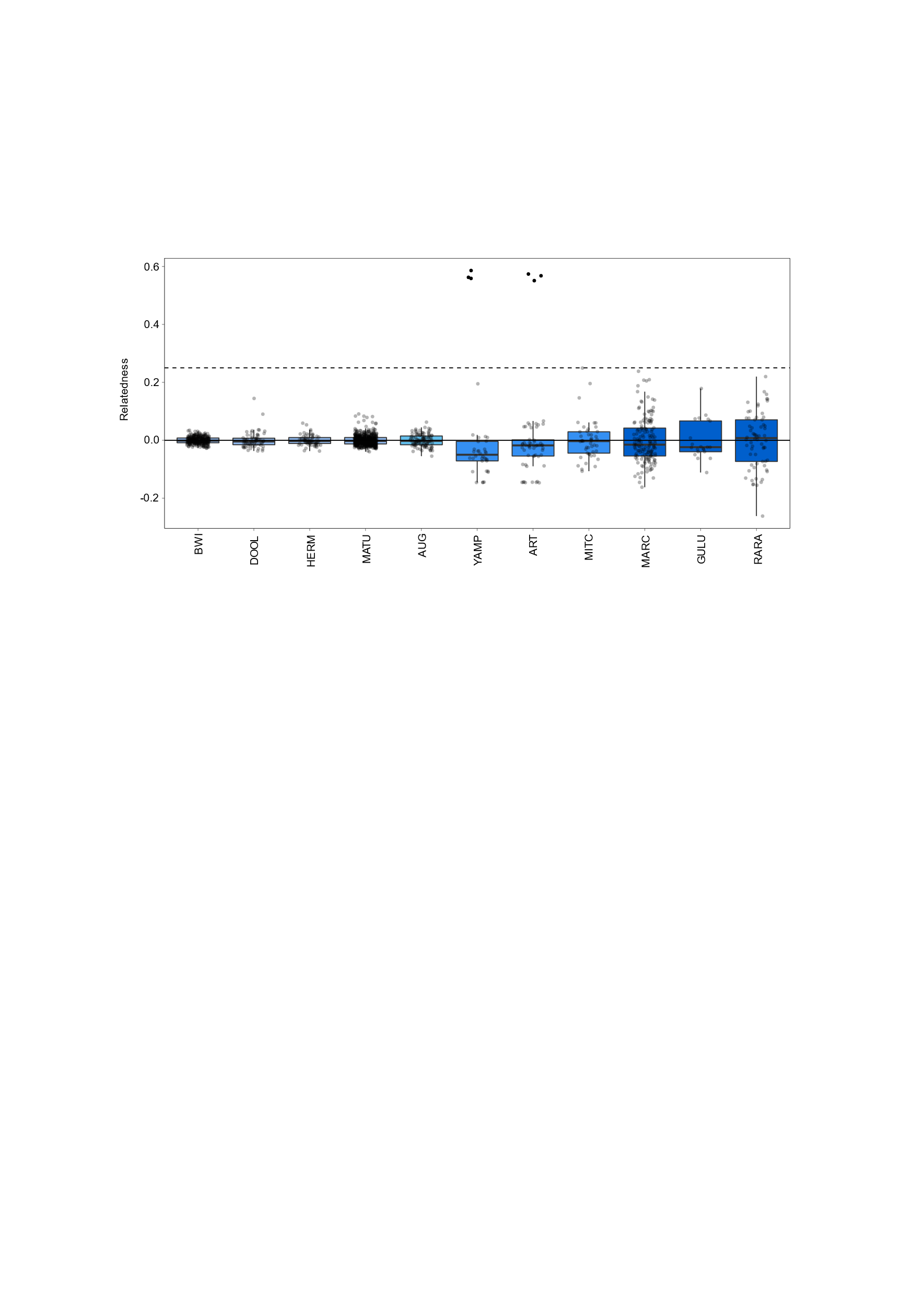


Figure S4. Pairwise related values for each population of golden bandicoot (*Isoodon auratus*) where individuals with a value >0.25 were considered to be closely related (threshold illustrated by dotted line) and removed from downstream analyses.


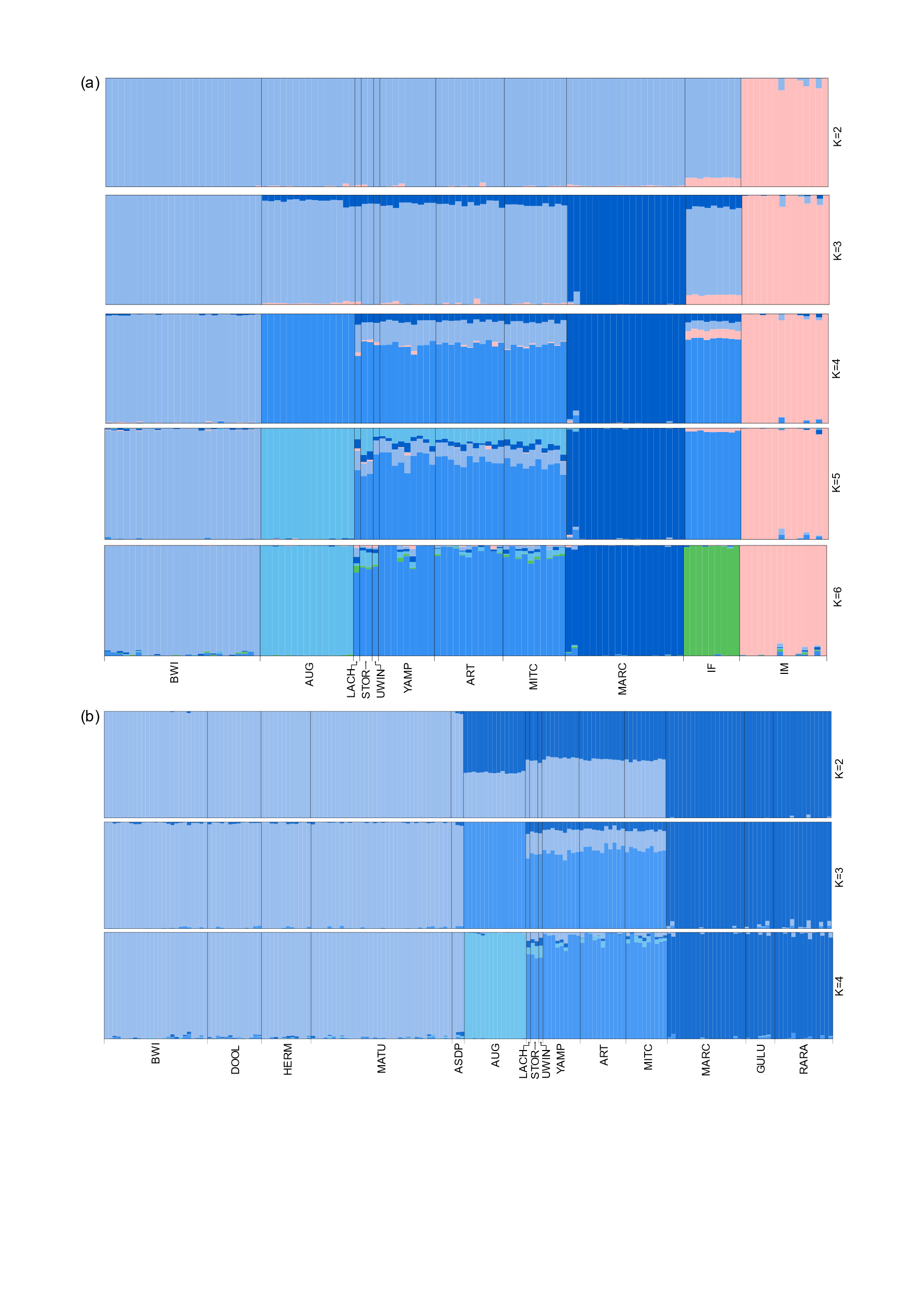


Figure S5. Patterns of hierarchical structuring when using all individuals in each population. Admixture coefficients were estimated using the ‘sNMF’ function in the R package LEA at different K values when using (a) the ‘*Isoodon*’ dataset and (b) the ‘*auratus*’ dataset. Populations include Barrow Island (BWI), Doole Island (DOOL), Hermite Island (HERM), Matuwa (MATU), Alice Springs Desert Park (ASDP), Augustus Island (AUG), Lachlan Island (LACH), Storr Island (STOR), Uwins Island (UWIN), Yampi Sound (YAMP), Artesian Range (ART), Mitchell Plateau (MITC), Marchinbar Island (MARC), Guluwuru Island (GULU), Raragala Island (RARA), *I. macrourus* (IM) and *I. fusciventer* (IF).


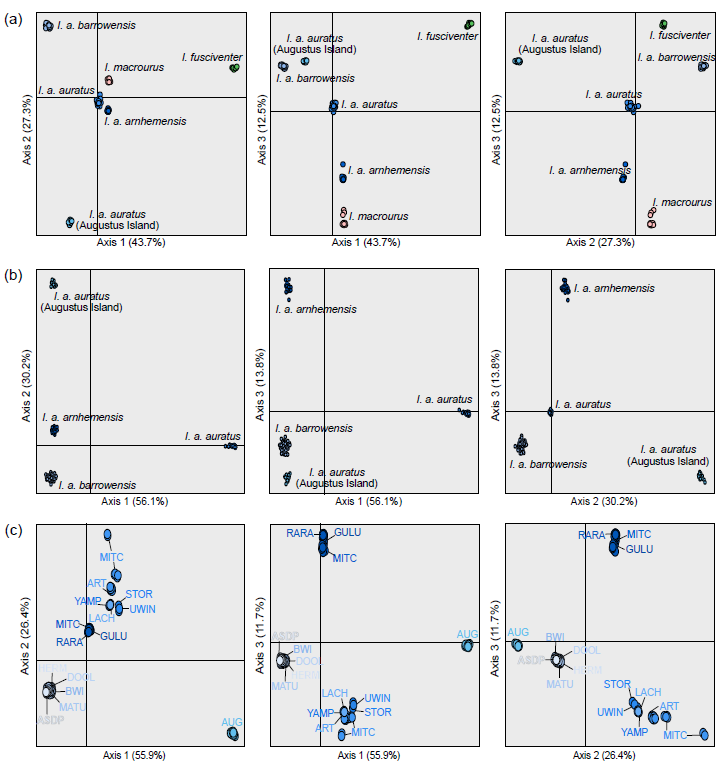


Figure S6. Discriminant Analysis of Principal Components (DAPC) of golden bandicoots (*Isoodon auratus*) when (a) ‘*Isoodon*’ dataset is used, (b) ‘*auratus*’ dataset is used with K=4, and (c) ‘*auratus*’ dataset is used with K=15. Populations include Barrow Island (BWI), Doole Island (DOOL), Hermite Island (HERM), Matuwa (MATU), Alice Springs Desert Park (ASDP), Augustus Island (AUG), Lachlan Island (LACH), Storr Island (STOR), Uwins Island (UWIN), Yampi Sound (YAMP), Artesian Range (ART), Mitchell Plateau (MITC), Marchinbar Island (MARC), Guluwuru Island (GULU) and Raragala Island (RARA).


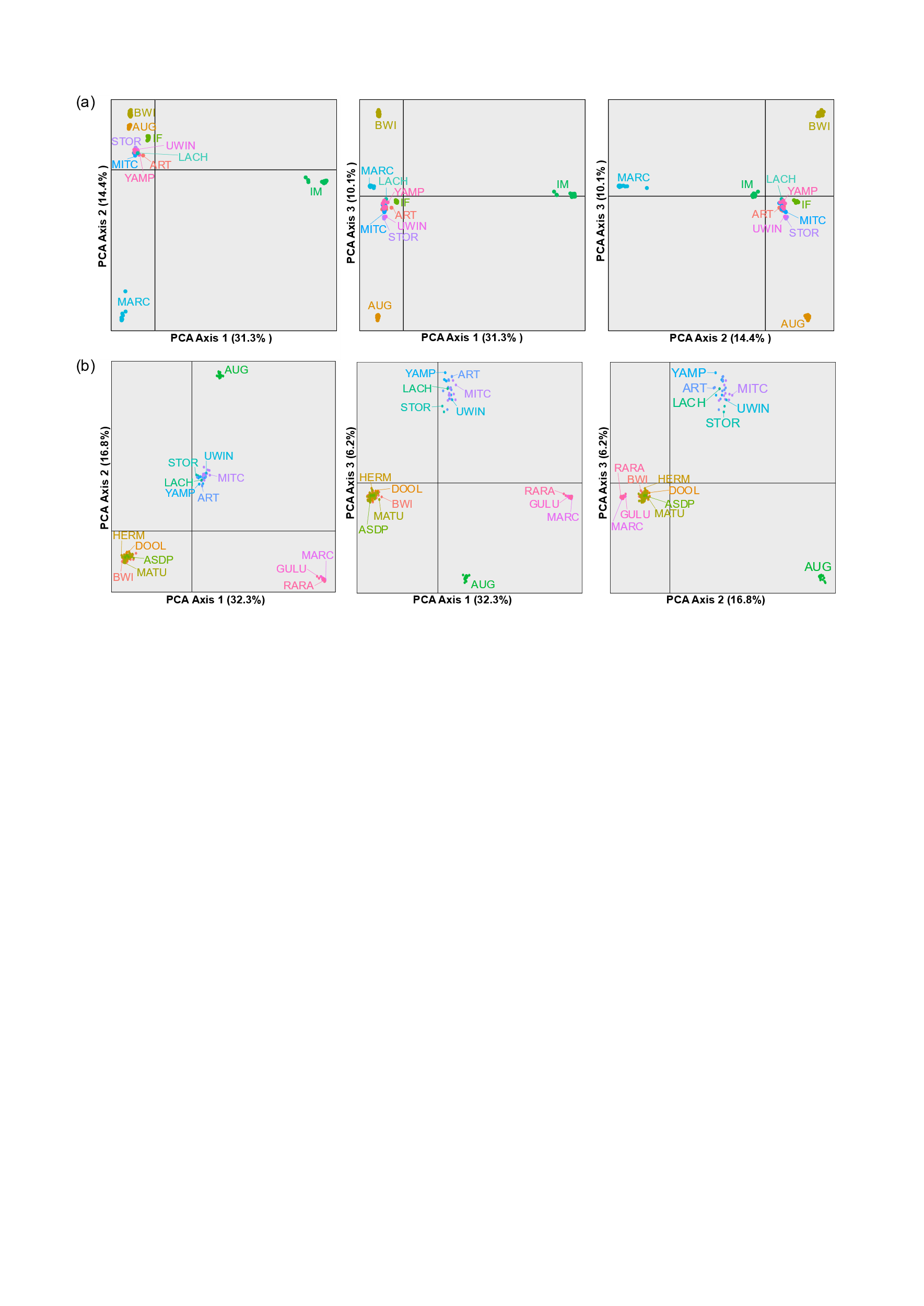


Figure S7. Principal coordinates analysis (PCoA) of golden bandicoots (*Isoodon auratus*) using (a) the ‘*Isoodon*’ dataset and (b) the ‘*auratus*’ dataset. Populations include Barrow Island (BWI), Doole Island (DOOL), Hermite Island (HERM), Matuwa (MATU), Alice Springs Desert Park (ASDP), Augustus Island (AUG), Lachlan Island (LACH), Storr Island (STOR), Uwins Island (UWIN), Yampi Sound (YAMP), Artesian Range (ART), Mitchell Plateau (MITC), Marchinbar Island (MARC), Guluwuru Island (GULU), Raragala Island (RARA), *I. macrourus* (IM) and *I. fusciventer* (IF).


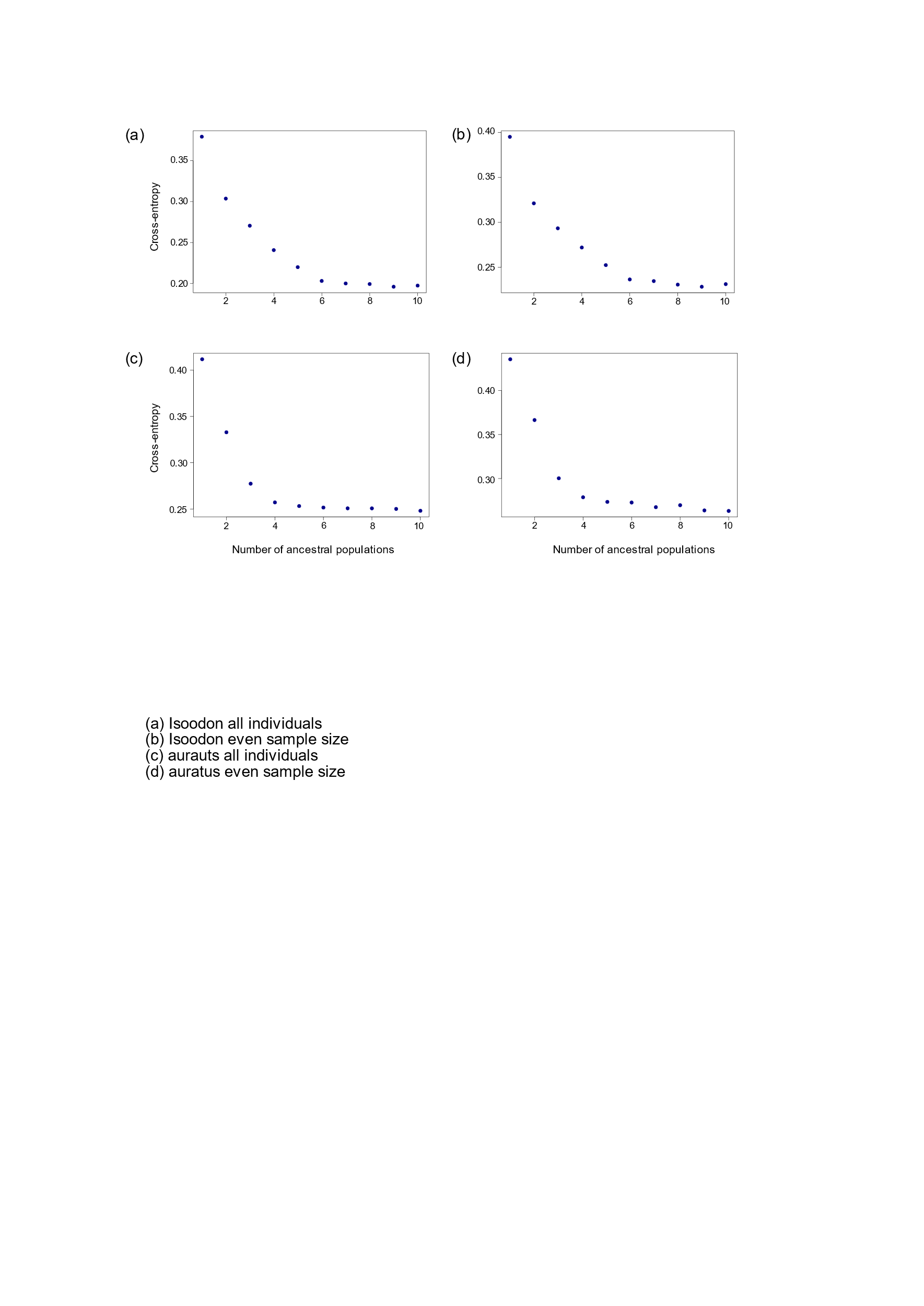


‘*Isoodon*’ dataset

(all individuals)

‘*Isoodon*’ dataset

(subsetted)

‘*auratus*’ dataset

(subsetted)

‘*auratus*’ dataset

(all individuals)

Figure S8. Cross-entropy plot used in R package LEA to identify hierarchical population structuring in golden bandicoots (*Isoodon auratus*) when (a) ‘*Isoodon*’ dataset was used with all individuals retained, (b) ‘*Isoodon*’ dataset where each population was subsetted to contain a maximum of 10 individuals, (c) ‘*auratus*’ dataset used with all individuals retained, and (d) ‘*auratus*’ dataset where each population was subsetted to contain a maximum of 10 individuals. Lower values of the cross-entropy criterion indicate a better fit to the data. A large drop in cross-entropy from the preceding point also indicates that a K value is well-supported.


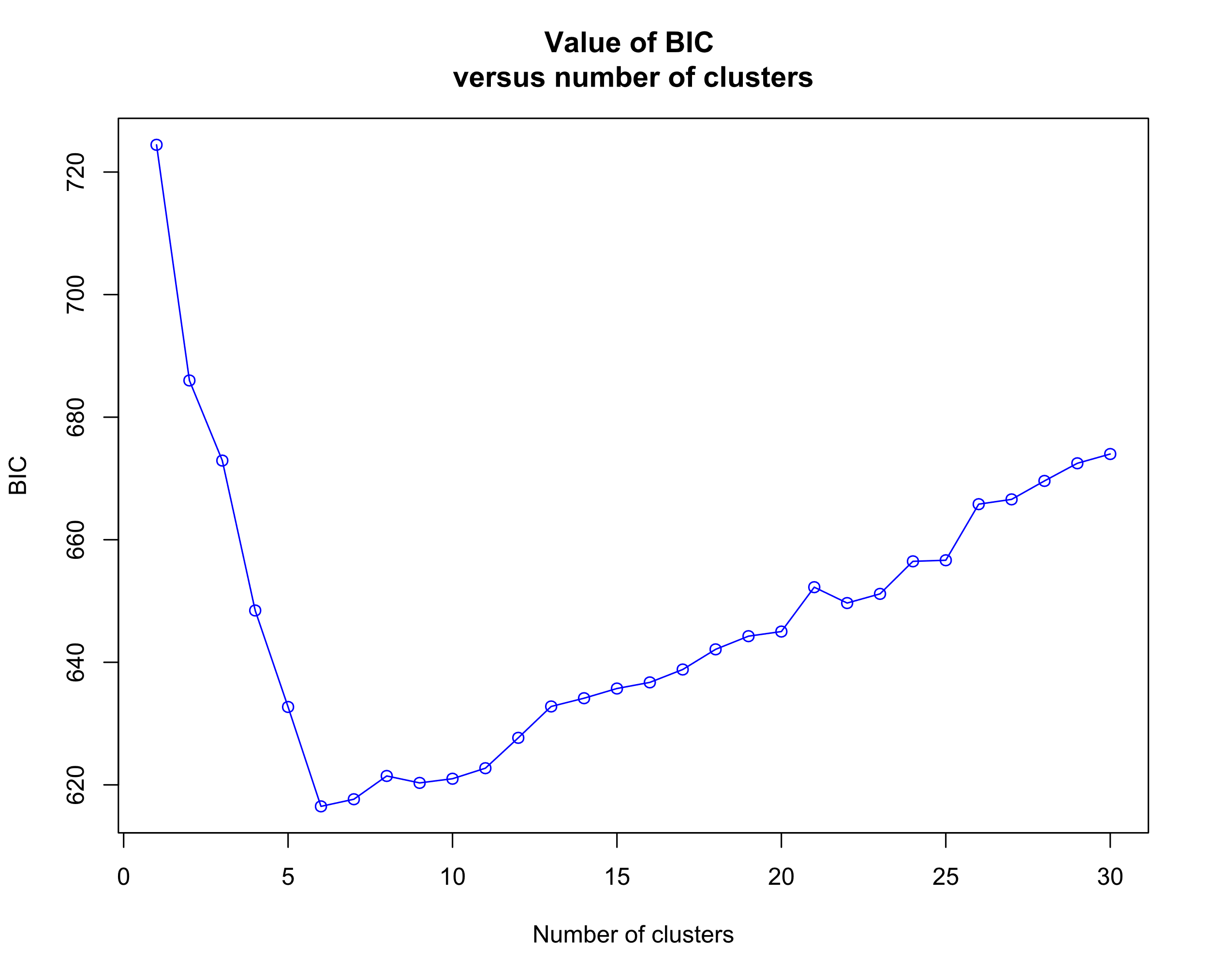

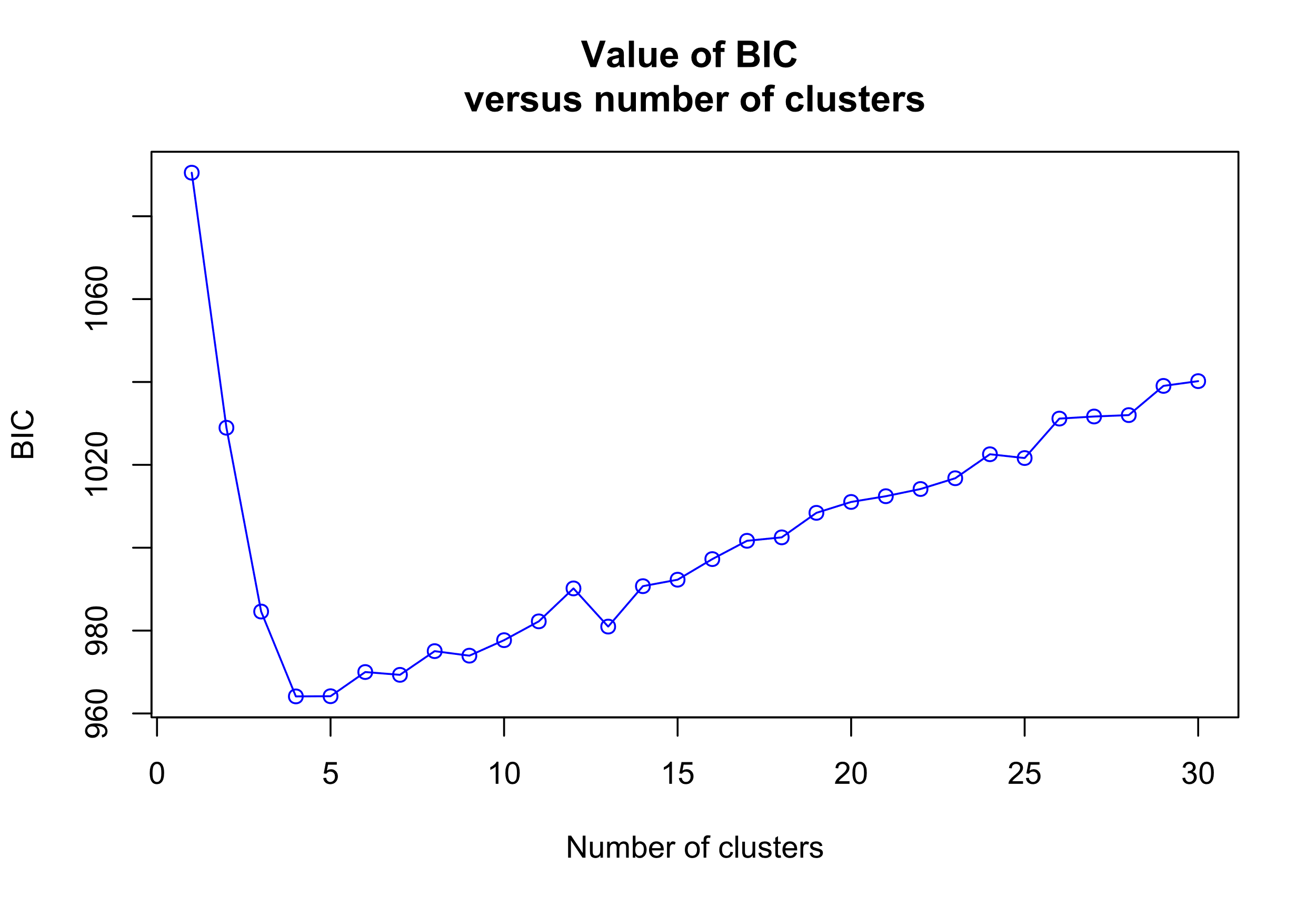


Figure S9. Bayesian Information Criterion (BIC) used to identify best-supported K value for the Discriminant Analysis of Principal Components (DAPC) for the ‘*Isoodon*’ dataset (top) and ‘*auratus*’ dataset (bottom). Values with the lowest BIC score indicate the best fit to the data.


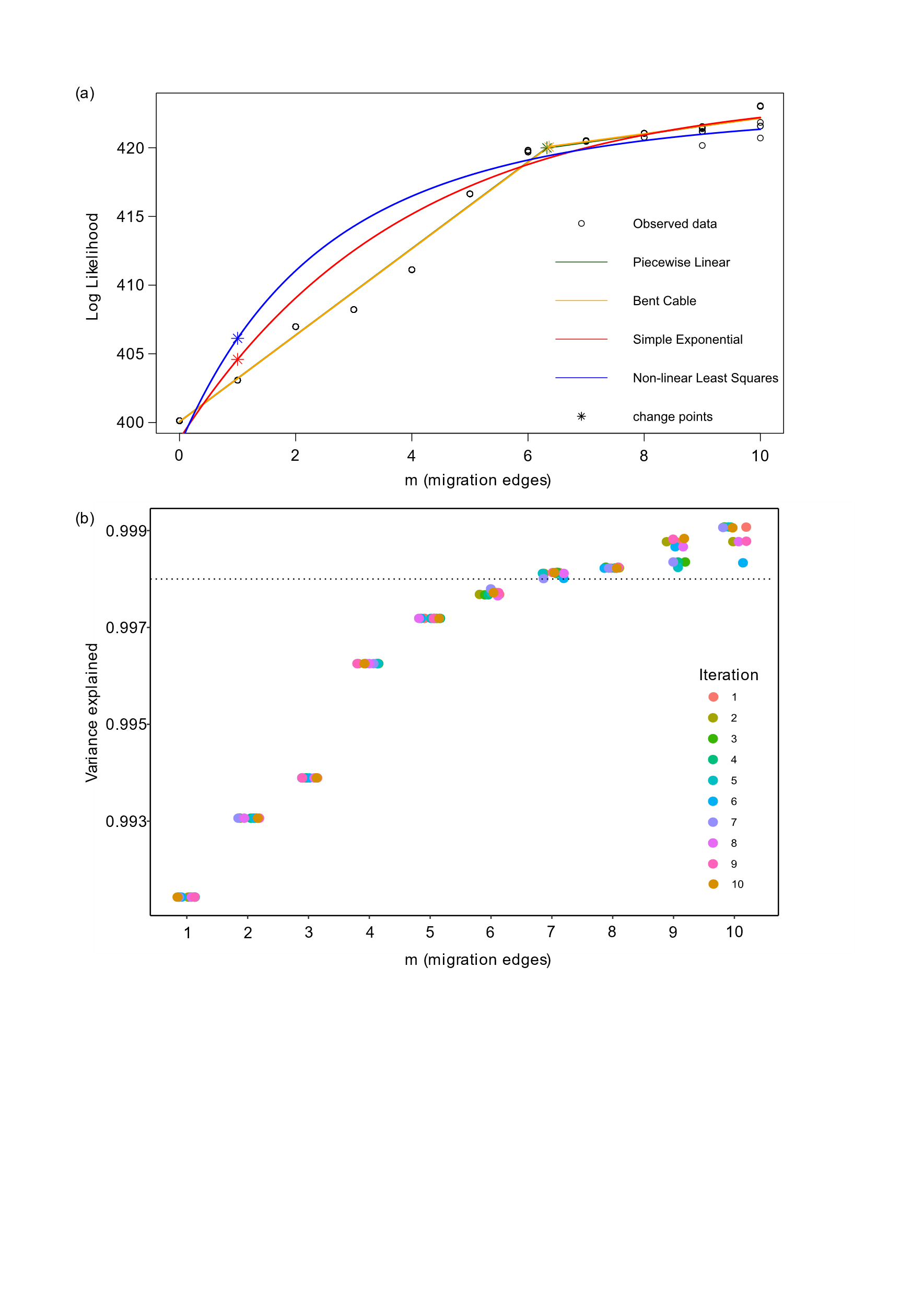


Figure S10. Determination of the optimal number of migration edges between 0 and 10 in TreeMix using (a) parametric models to the log likelihood values across runs and compare them to the Akaike information criterion, where each change point indicates a potential migration event, and (b) the percentage of variance explained by each migration event where the dotted line represents the 99.8% threshold recommended by Pickrell & Pritchard (2012).


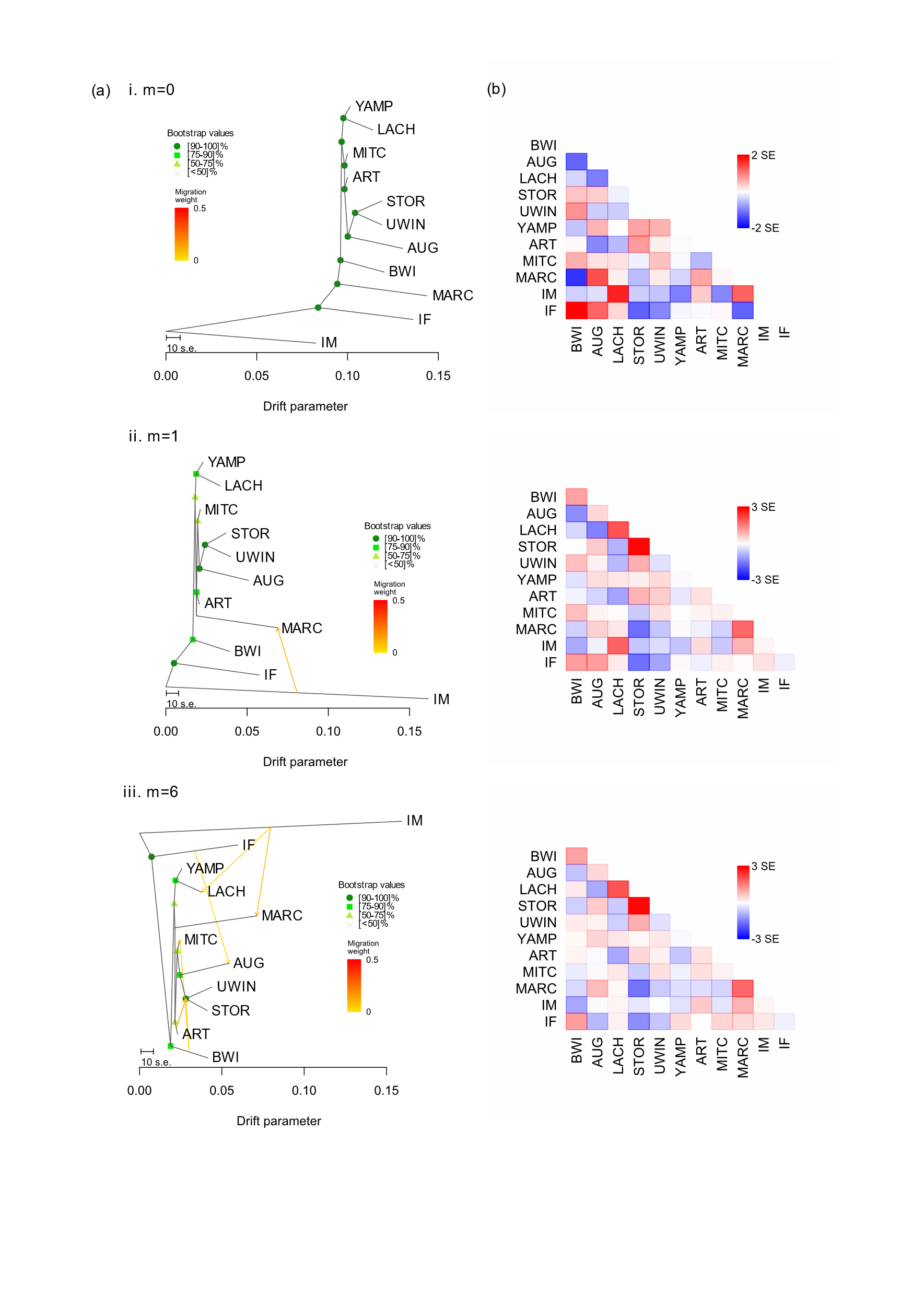


Figure S11. (a) TreeMix consensus tree and bootstrap values display the relationships among populations as a bifurcating maximum-likelihood tree for (i) no migration edge (m=0), (ii) one migration edge (m=1) and (iii) six migration edges (m=6). Horizontal branch lengths represent the amount of genetic drift that has occurred along each branch. (b) Residual fit of the observed versus the predicted squared allele frequency difference, expressed as the number of SE of the deviation. SE values are represented by colours according to the palette on the right. Residuals above zero indicate populations that are more closely related to each other in the data than in the best-fit tree and have potentially undergone admixture. Negative residuals represent populations that are less closely related in the data than represented in the best-fit tree. Populations include Barrow Island (BWI), Doole Island (DOOL), Hermite Island (HERM), Matuwa (MATU), Alice Springs Desert Park (ASDP), Augustus Island (AUG), Lachlan Island (LACH), Storr Island (STOR), Uwins Island (UWIN), Yampi Sound (YAMP), Artesian Range (ART), Mitchell Plateau (MITC), Marchinbar Island (MARC), Guluwuru Island (GULU), Raragala Island (RARA), *I. macrourus* (IM) and *I. fusciventer* (IF).


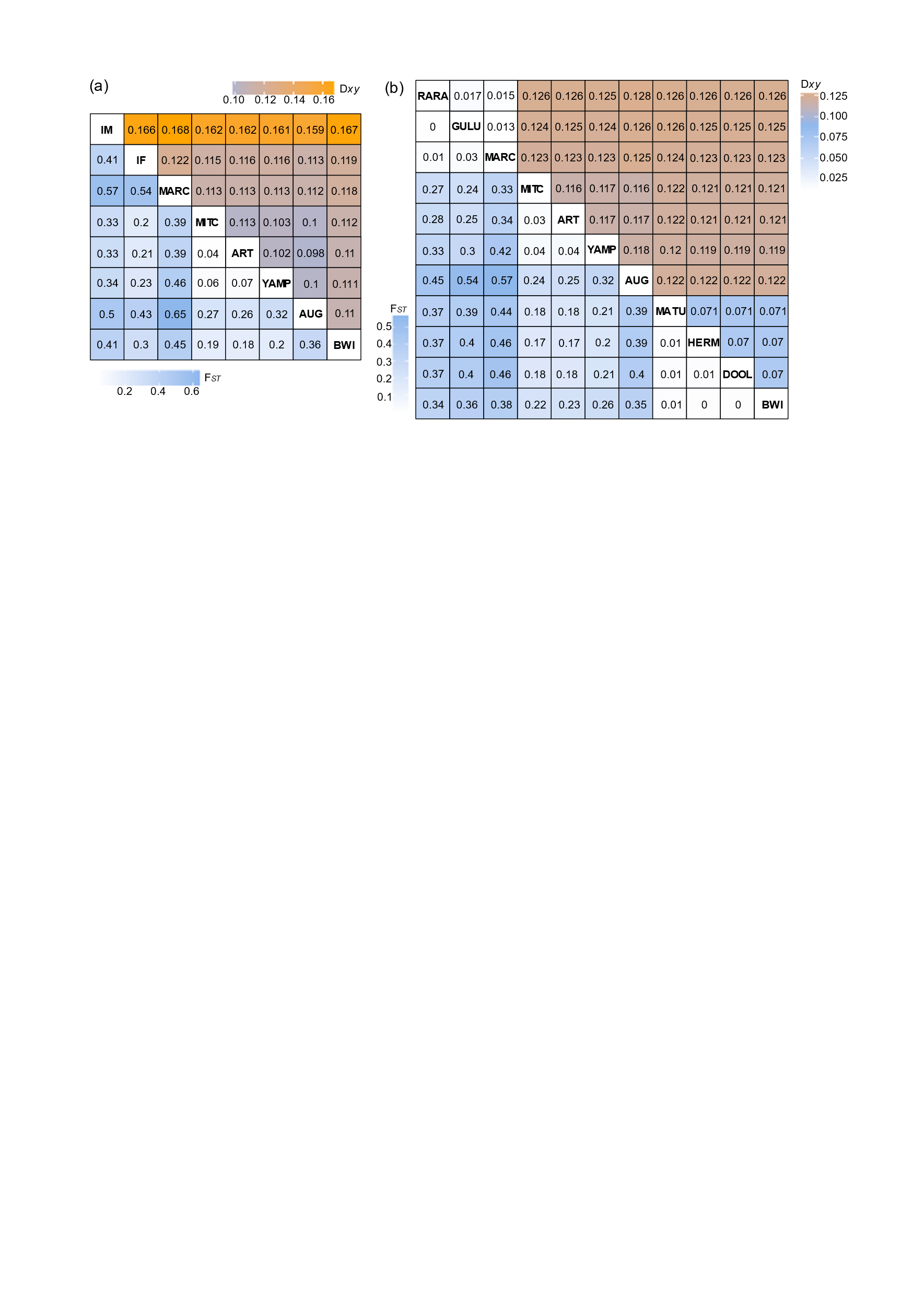


Figure S12. Heatmap of pairwise F_ST_ values between each population (lower triangle) and pairwise D_XY_ values (upper triangle) for (a) the ‘*Isoodon*’ dataset and (b) the ‘*auratus*’ dataset. Pairwise F_ST_ and D_XY_ values are represented by colours according to their associated colour palette. Populations include Barrow Island (BWI), Doole Island (DOOL), Hermite Island (HERM), Matuwa (MATU), Alice Springs Desert Park (ASDP), Augustus Island (AUG), Lachlan Island (LACH), Storr Island (STOR), Uwins Island (UWIN), Yampi Sound (YAMP), Artesian Range (ART), Mitchell Plateau (MITC), Marchinbar Island (MARC), Guluwuru Island (GULU), Raragala Island (RARA), *I. macrourus* (IM) and *I. fusciventer* (IF).


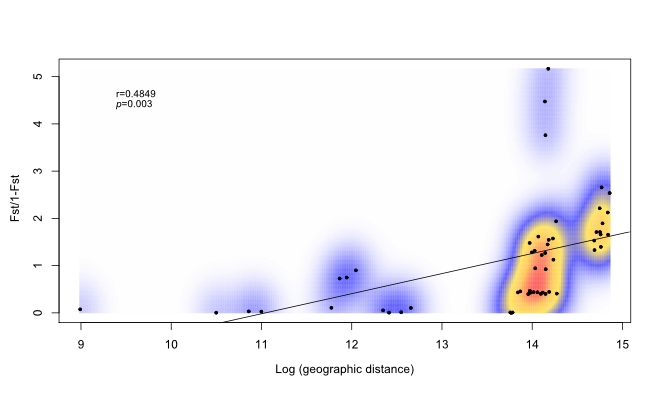


Figure S13. Mantel test of isolation by distance amongst *Isoodon auratus* populations using the ‘auratus’ dataset. Each point represents one population pairwise F_sT_/(1-F_ST_) plotted against logged Euclidean distance between paired populations.


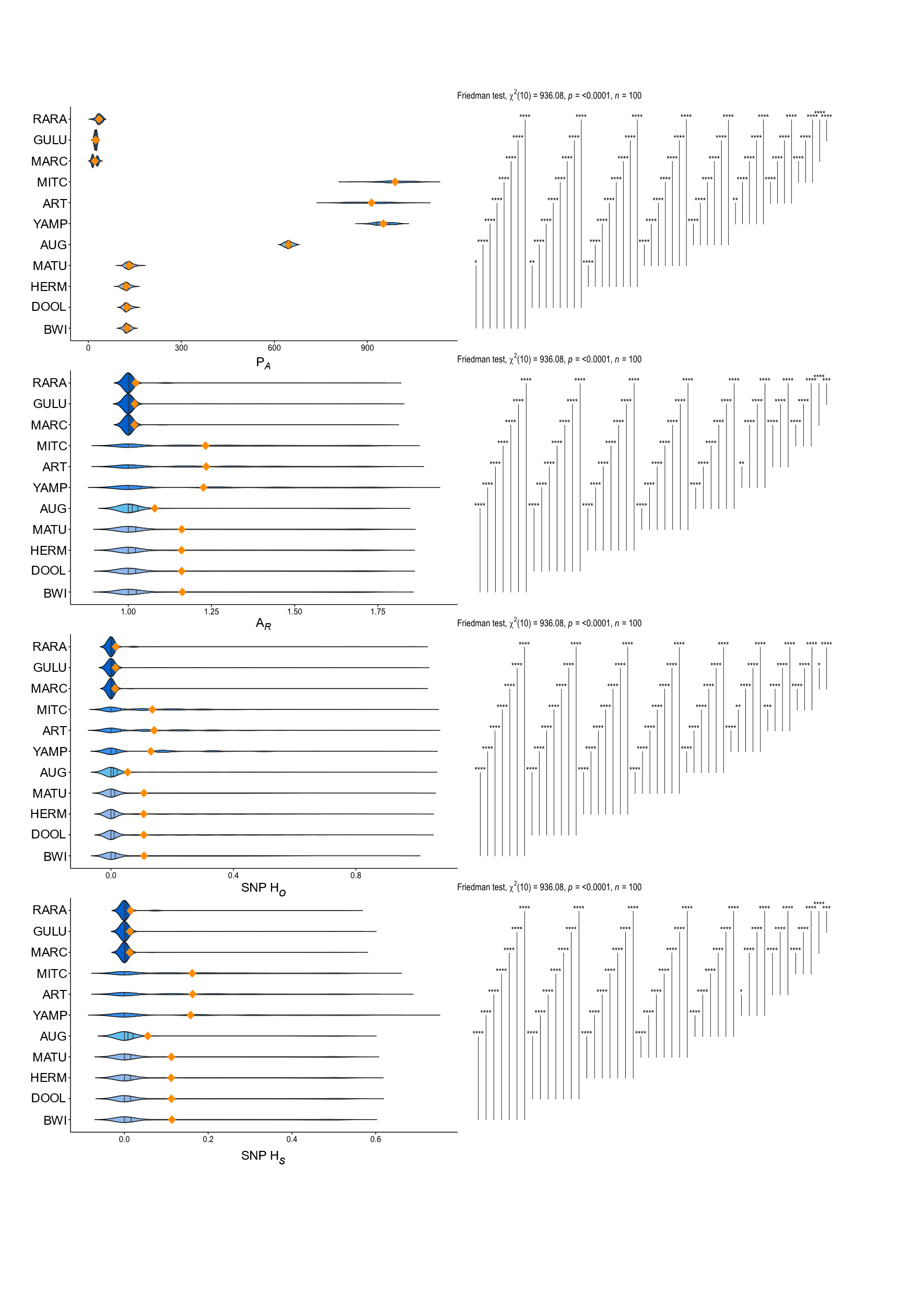

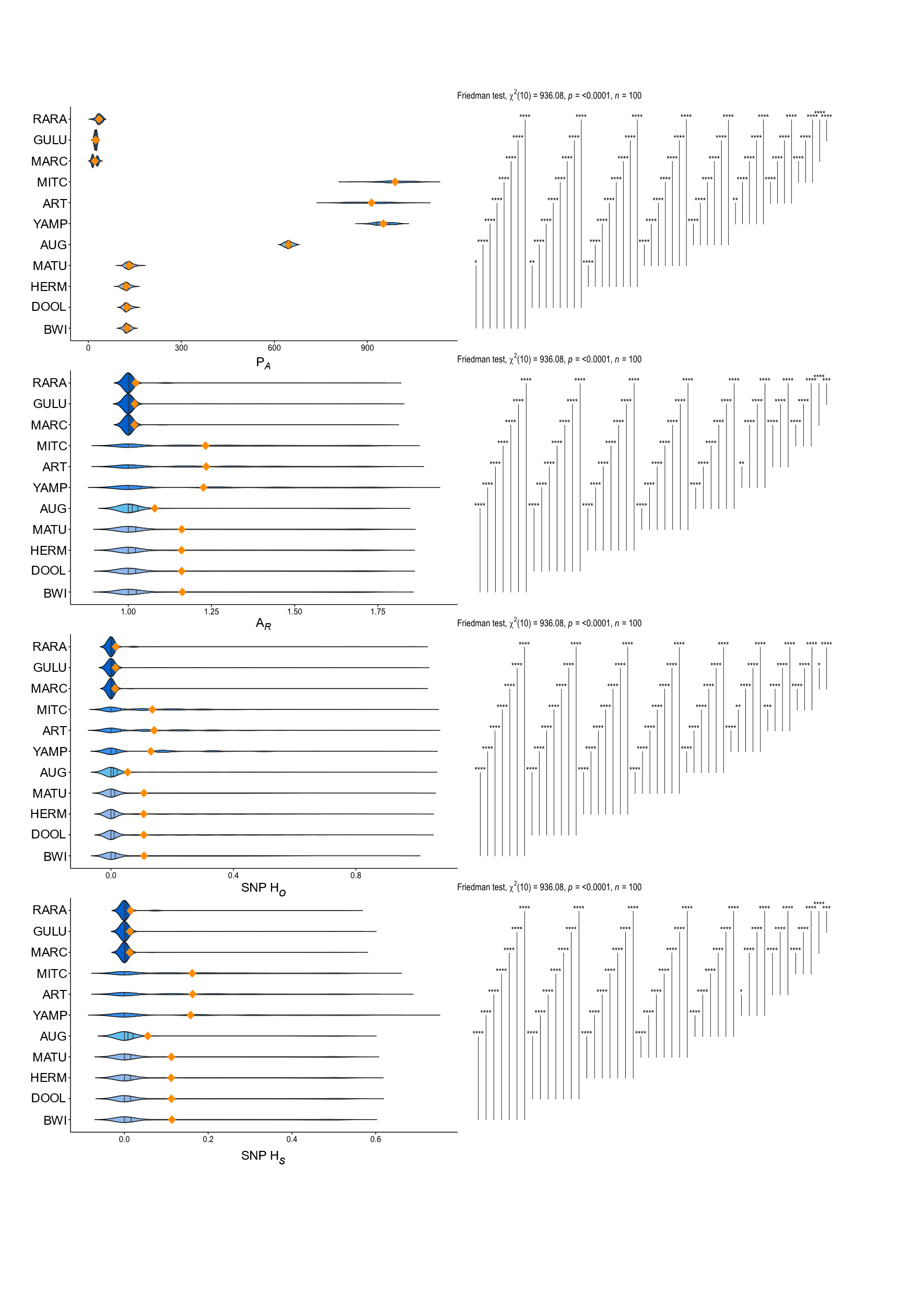


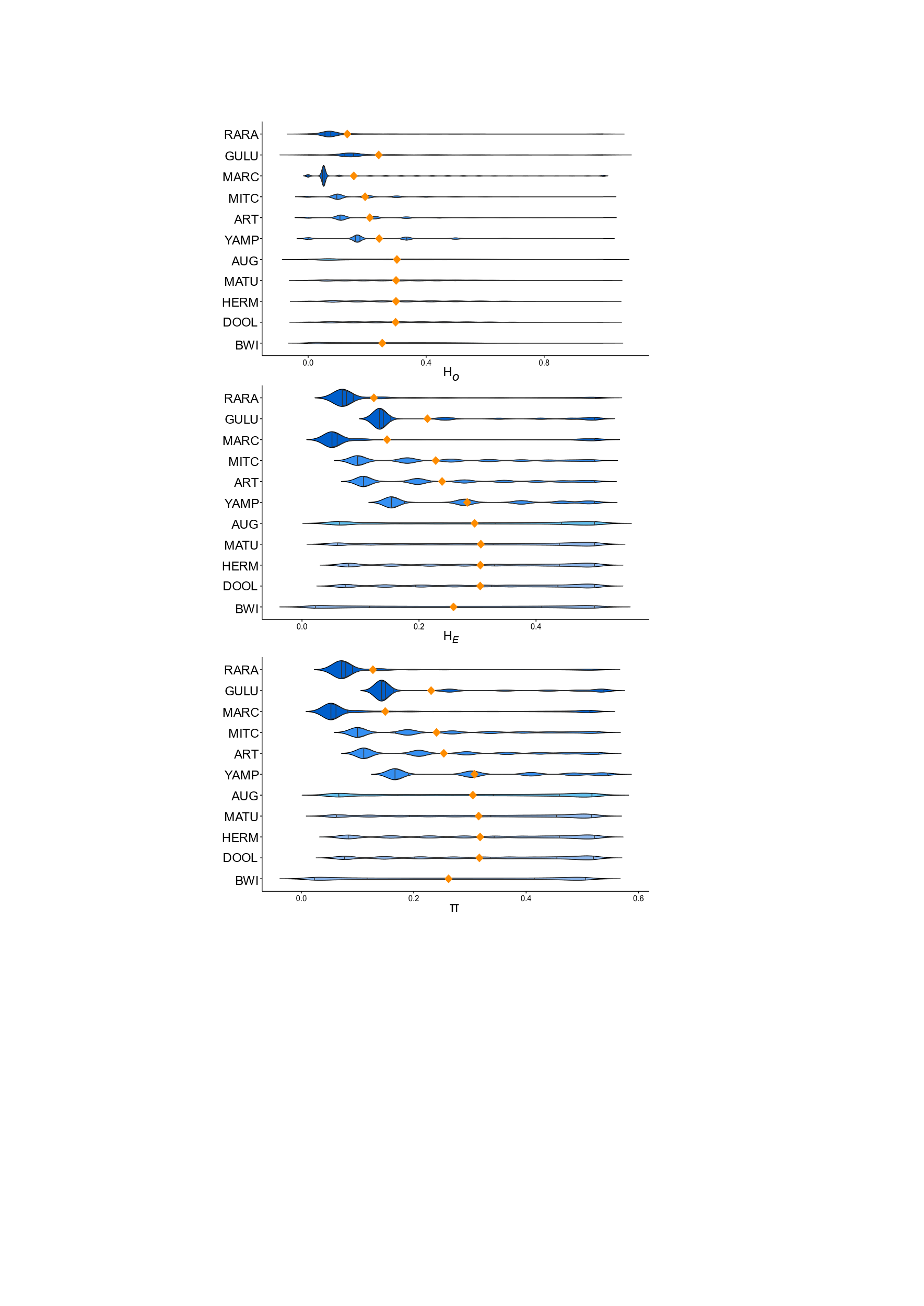

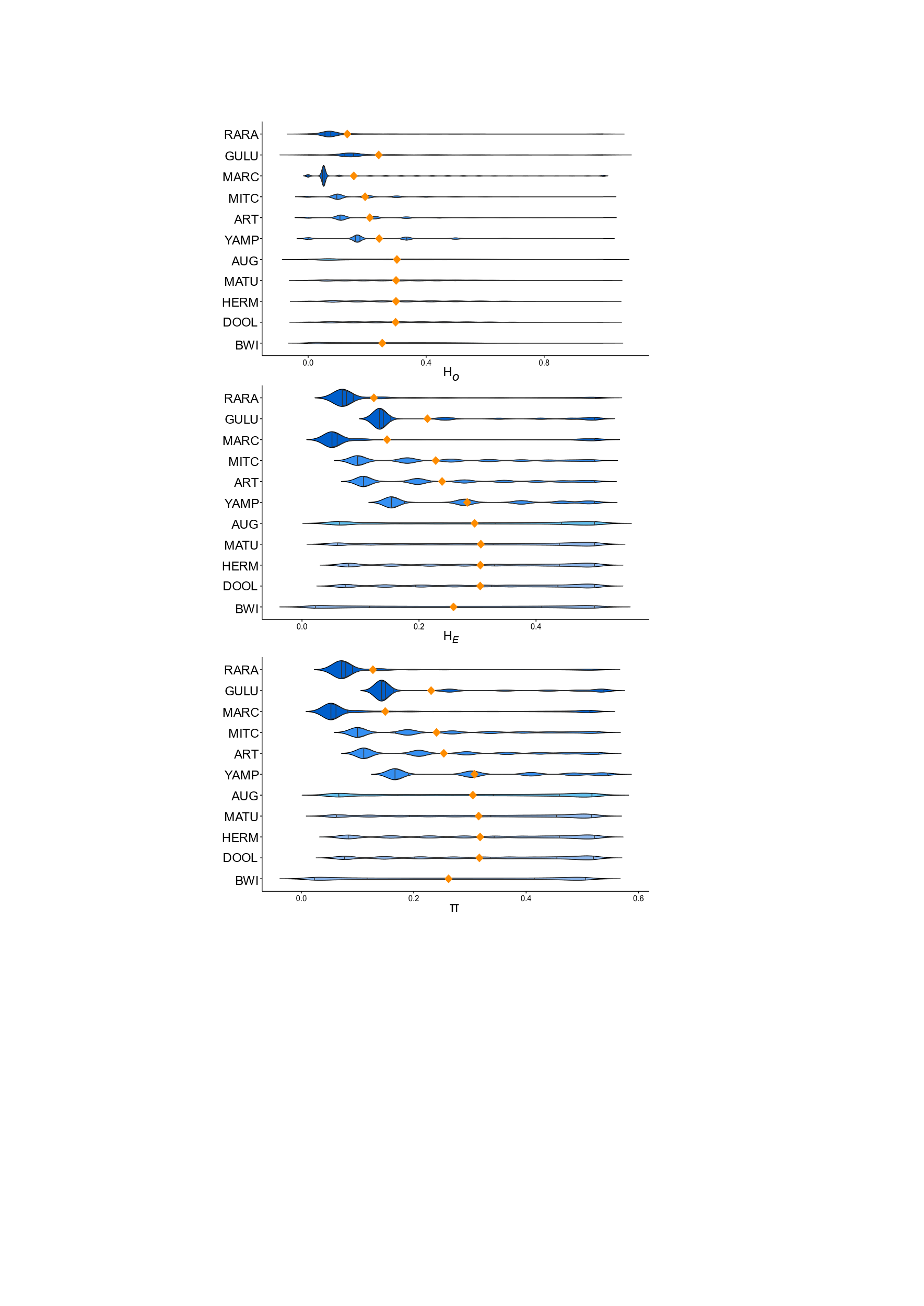


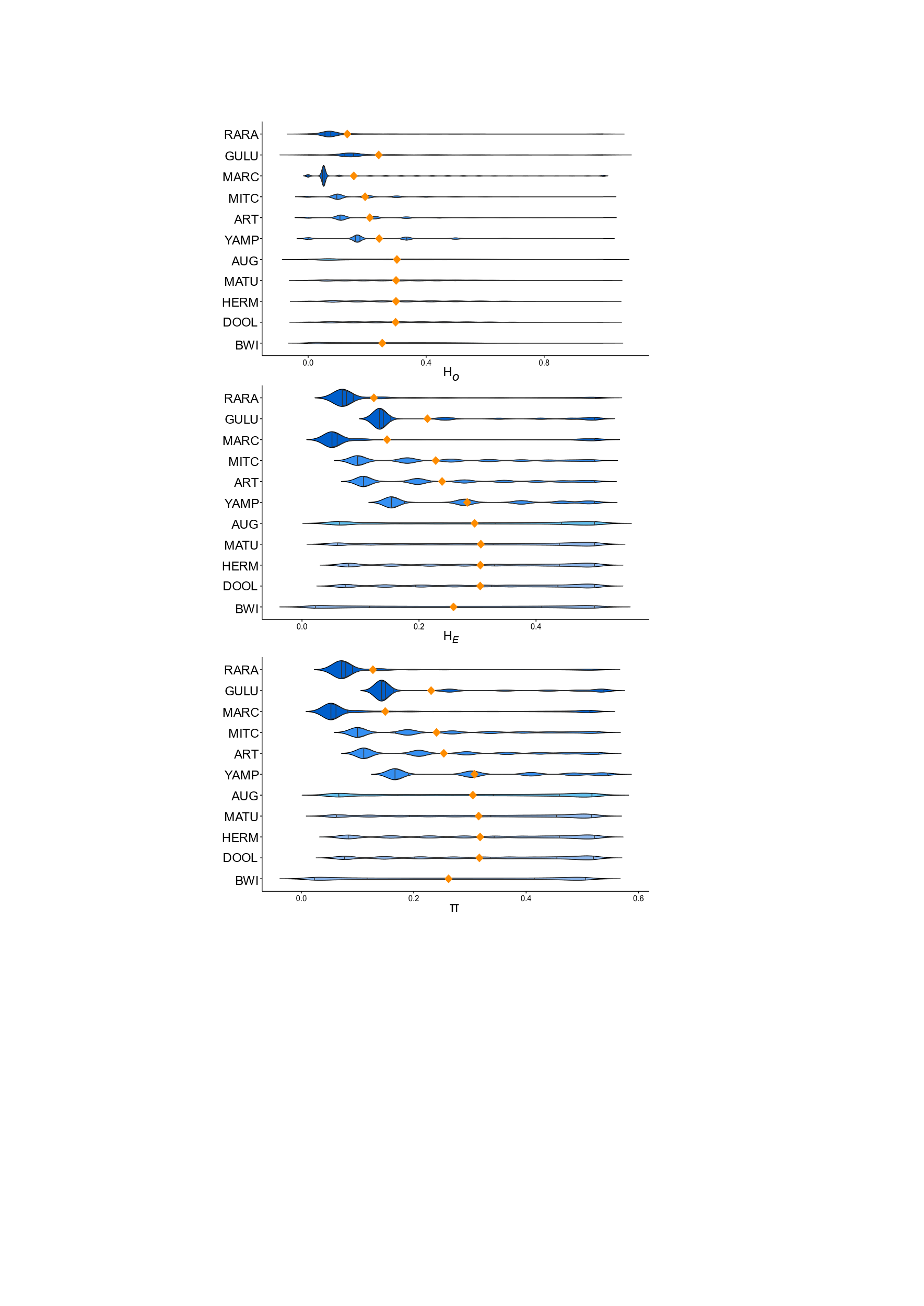


Figure S14 . Violin plots of diversity metrics across each golden bandicoot (*Isoodon auratus*) population; private alleles (P_A_), allelic richness (A_R_), observed heterozygosity (H_O_), expected heterozygosity (H_E_), and nucleotide diversity (π). P_A_ and A_R_ were calculated using 8244 SNPs called across all populations. H_O_, H_E_, and π were extracted from populations in Stacks v. 2.2 (Catchen et al., 2013) summary statistics output when SNPs were called for each population independently. Quantiles are shown on the violin plot and the mean is represented by an orange diamond. Populations include Barrow Island (BWI), Doole Island (DOOL), Hermite Island (HERM), Matuwa (MATU), Alice Springs Desert Park (ASDP), Augustus Island (AUG), Lachlan Island (LACH), Storr Island (STOR), Uwins Island (UWIN), Yampi Sound (YAMP), Artesian Range (ART), Mitchell Plateau (MITC), Marchinbar Island (MARC), Guluwuru Island (GULU) and Raragala Island (RARA).

Table S2. Temporal population-level diversity metrics in translocated populations calculated for datasets where SNPs were independently called for each population. Parameters include allelic richness (A_R­_), number of SNPs when each translocated population has been called independently, observed heterozygosity (H_O_), expected heterozygosity (H_E_), nucleotide diversity (π), and the number of variant sites when each population is called independently. Standard errors are shown in parentheses, except variant sites where the total number of sites called (variant and invariant) are stated in parentheses. Populations include Doole Island (DOOL), Hermite Island (HERM), Matuwa (MATU), Guluwuru Island (GULU) and Raragala Island (RARA).

|  |  | **n** | **A_R_** | **SNPs** | **H_O_** (×100) | **H_E_** (×100) | **π** (×100) | **Variant sites**  **(Total sites)** |
| --- | --- | --- | --- | --- | --- | --- | --- | --- |
| **DOOL** | 2011 | 12 | 1.978 (0.001) | 16,719 | 0.189 (0.001) | 0.194 (0.001) | 0.203 (0.001) | 28,850  (4,116,444) |
|  | 2019 | 13 | 1.974 (0.001) |  | 0.190 (0.001) | 0.192 (0.001) | 0.200 (0.001) | 28,850  (4,116,629) |
| **HERM** | 2010 | 12 | 1.981 (0.001) | 17,078 | 0.181 (0.001) | 0.184 (0.001) | 0.192 (0.001) | 30,108  (4,479,067) |
|  | 2019 | 12 | 1.978 (0.001) |  | 0.180 (0.001) | 0.181 (0.001) | 0.189 (0.001) | 30,108  (4,478,871) |
| **MATU** | 2009 | 18 | 1.977 (0.001) | 17,843 | 0.199 (0.001) | 0.202 (0.001) | 0.208 (0.001) | 26,209  (3,622,188) |
|  | 2019 | 16 | 1.967 (0.001) |  | 0.197 (0.002) | 0.198 (0.001) | 0.205 (0.001) | 26,209  (3,621,941) |
| **GULU** | 2009 | 3 |  |  | 0.042 (0.001) | 0.033 (0.0001) | 0.04 (0.001) | 10,700  (5,323,562) |
|  | 2011 | 4 |  |  | 0.053 (0.001) | 0.045 (0.001) | 0.051 (0.001) | 10,700  (5,324,201) |

Table S3. Optimisation scenarios for the conservation of alleles in the golden bandicoot (*Isoodon auratus*) indicating the proportion of times that each population was chosen in a given scenario from 100 iterations of randomly sampling four individuals per location and counting the number of alleles. Populations include Barrow Island (BWI), Doole Island (DOOL), Hermite Island (HERM), Matuwa (MATU), Alice Springs Desert Park (ASDP), Augustus Island (AUG), Lachlan Island (LACH), Storr Island (STOR), Uwins Island (UWIN), Yampi Sound (YAMP), Artesian Range (ART), Mitchell Plateau (MITC), Marchinbar Island (MARC), Guluwuru Island (GULU) and Raragala Island (RARA).

| No. of populations conserved | **BWI** | **DOOL** | **HERM** | **MATU** | **AUG** | **YAMP** | **ART** | **MITC** | **MARC** | **GULU** | **RARA** |
| --- | --- | --- | --- | --- | --- | --- | --- | --- | --- | --- | --- |
| **1** | 0 | 0 | 0 | 0 | 0 | 0 | 67 | 33 | 0 | 0 | 0 |
| **2** | 5 | 0 | 1 | 0 | 0 | 62 | 66 | 66 | 0 | 0 | 0 |
| **3** | 53 | 4 | 27 | 16 | 0 | 59 | 73 | 68 | 0 | 0 | 0 |
| **4** | 53 | 6 | 24 | 17 | 0 | 100 | 100 | 100 | 0 | 0 | 0 |
| **5** | 54 | 4 | 25 | 17 | 0 | 100 | 100 | 100 | 36 | 9 | 55 |
| **6** | 54 | 6 | 28 | 13 | 0 | 100 | 100 | 100 | 39 | 6 | 55 |
| **7** | 64 | 43 | 45 | 48 | 0 | 100 | 100 | 100 | 39 | 13 | 48 |
| **8** | 81 | 81 | 54 | 84 | 0 | 100 | 100 | 100 | 35 | 21 | 44 |
| **9** | 100 | 100 | 100 | 100 | 0 | 100 | 100 | 100 | 33 | 27 | 40 |
| **10** | 100 | 100 | 100 | 100 | 0 | 100 | 100 | 100 | 15 | 96 | 89 |
| **11** | 100 | 100 | 100 | 100 | 0 | 100 | 100 | 100 | 82 | 100 | 100 |
